# Supplementary material for: Diffusion on PCA-UMAP Manifold: The Impact of Data Structure Preservation to Denoise High-Dimensional Single-Cell RNA Sequencing Data
Source: Biology (Basel). 2024 Jul 9;13(7):512. doi: 10.3390/biology13070512 (PMC11274112; doi:10.3390/biology13070512)

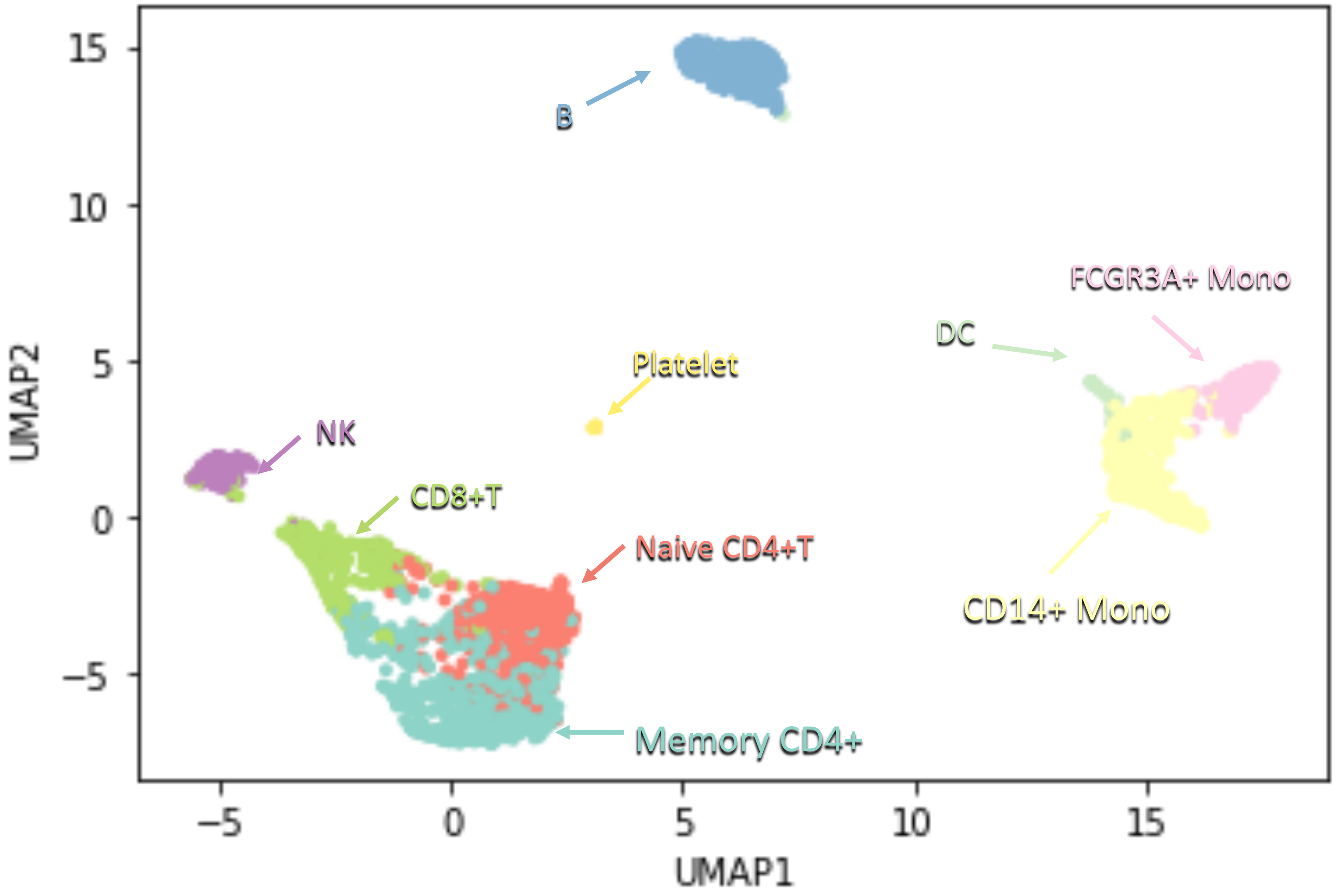

(A) **UMAP projection of the distinct PBMC cell phenotypes**

MAGIC imputation evaluation of knn (cell information sharing) with t = 5 pca= 30

sc-PHENIX imputation evaluation of knn (cell information sharing) with t = 5 pca= 30

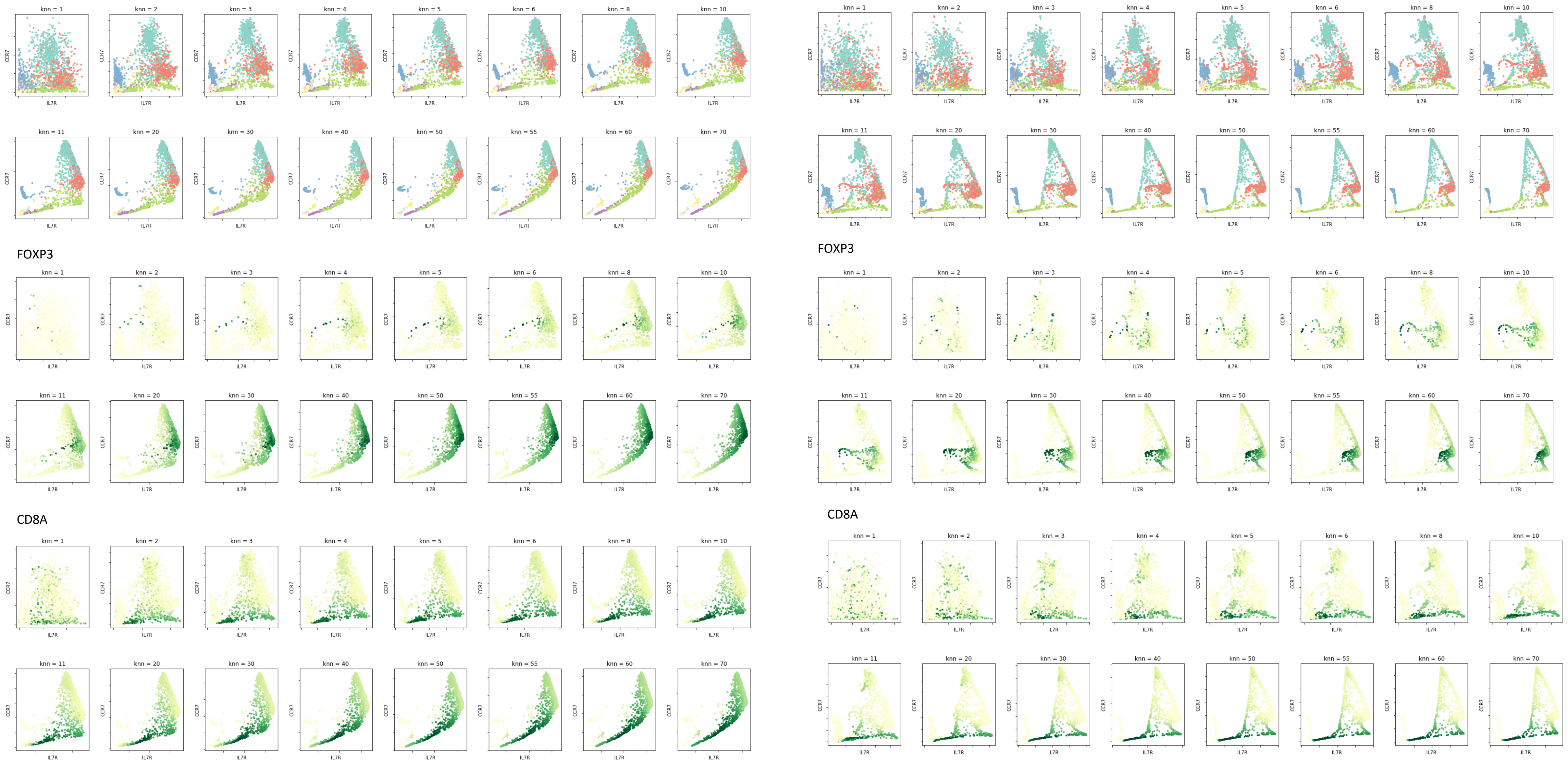

(B) Imputation evaluation of increasing values of knn with t = 5 (few random walks), pca= 30 using MAGIC and sc-PHENIX(PCA-UMAP space). Here, the CCR7-IL7R interaction visualizing the PBMC clusters, recovered gene expression of FOXP3 and CD8A.

MAGIC imputation evaluation of knn (cell information sharing) with t = 10 pca= 30

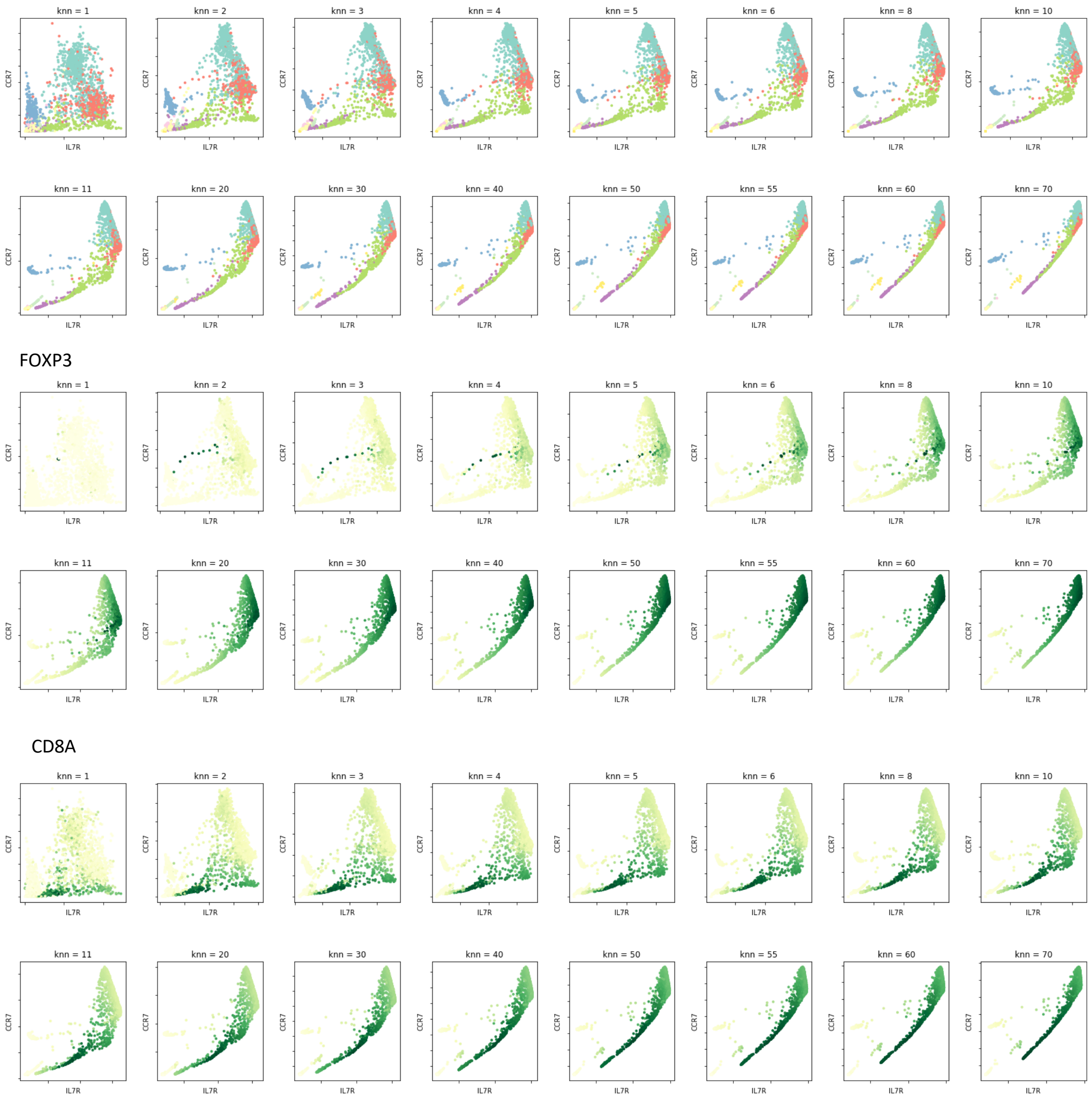

sc-PHENIX imputation evaluation of knn (cell information sharing) with t = 10 pca= 30

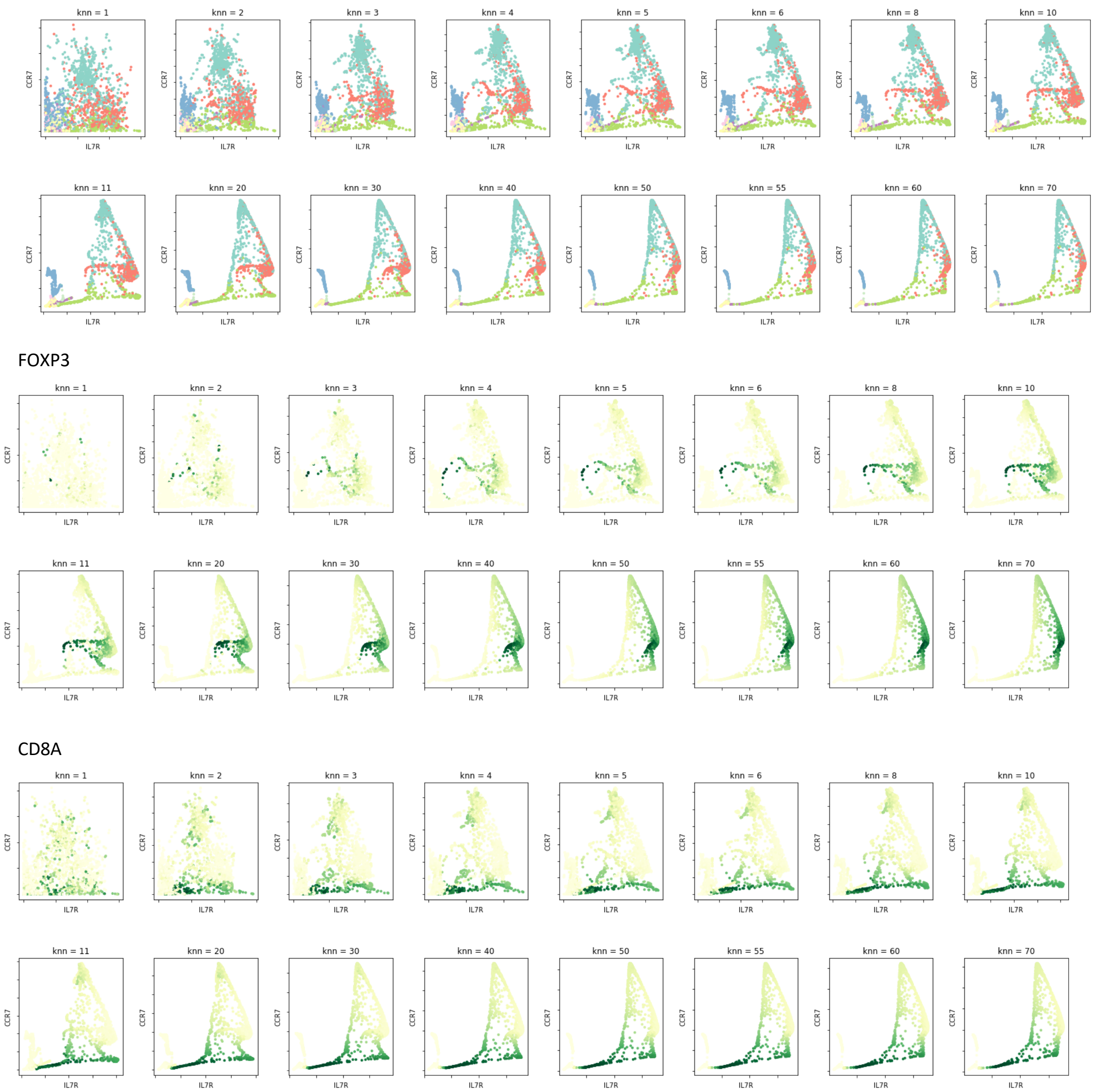

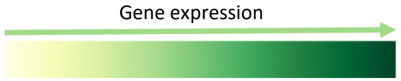

(C) Imputation evaluation of increasing values of knn with t = 10 (more random walks) pca= 30 using MAGIC and sc-PHENIX(PCA-UMAP space). Here, the CCR7-IL7R interaction visualizing the PBMC clusters, recovered gene expression of FOXP3 and CD8A.

MAGIC imputation evaluation of t (diffusion time) with knn= 5 pca= 30

sc-PHENIX imputation evaluation of t (diffusion time) with knn= 5 pca= 30

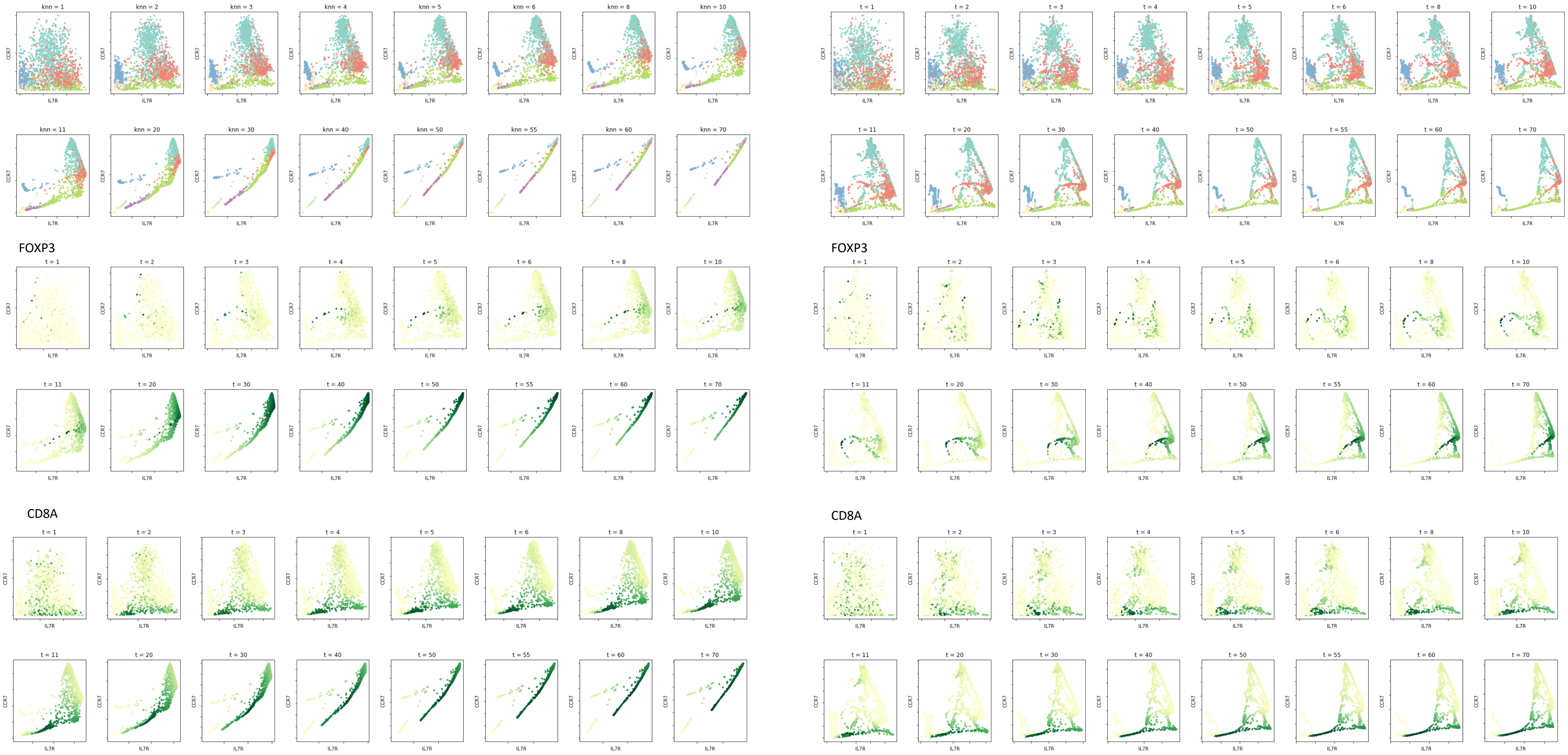

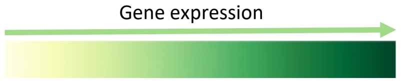

(D) Imputation evaluation of increasing values of t with knn=5 (less nearest neighbors) pca= 30 using MAGIC and sc-PHENIX(PCA-UMAP space). Here, the CCR7-IL7R interaction visualizing the PBMC clusters, recovered gene expression of FOXP3 and CD8A.

MAGIC imputation evaluation of t (diffusion time) with knn= 35 pca= 30

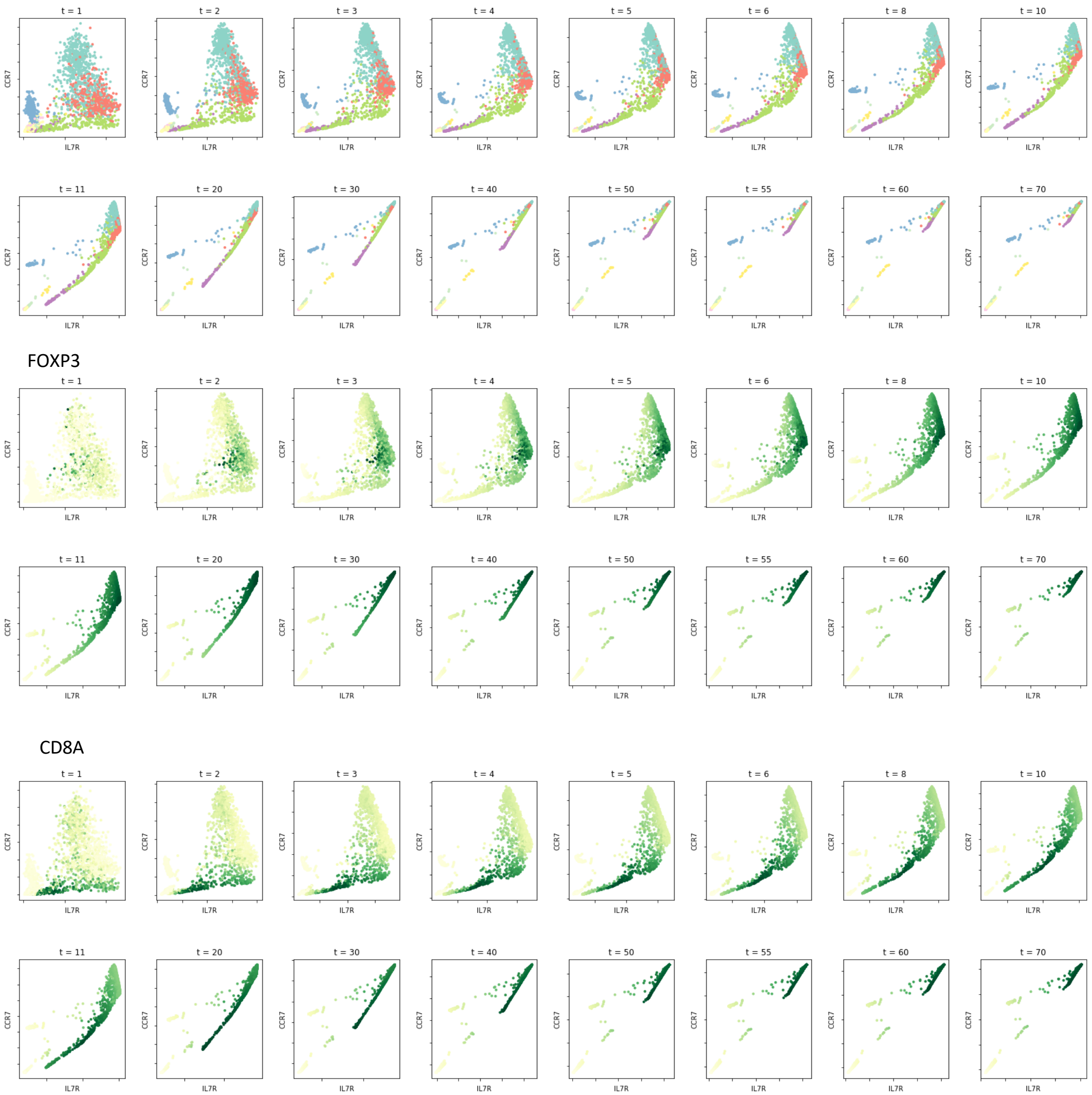

sc-PHENIX imputation evaluation of t (diffusion time) with knn= 35 pca= 30

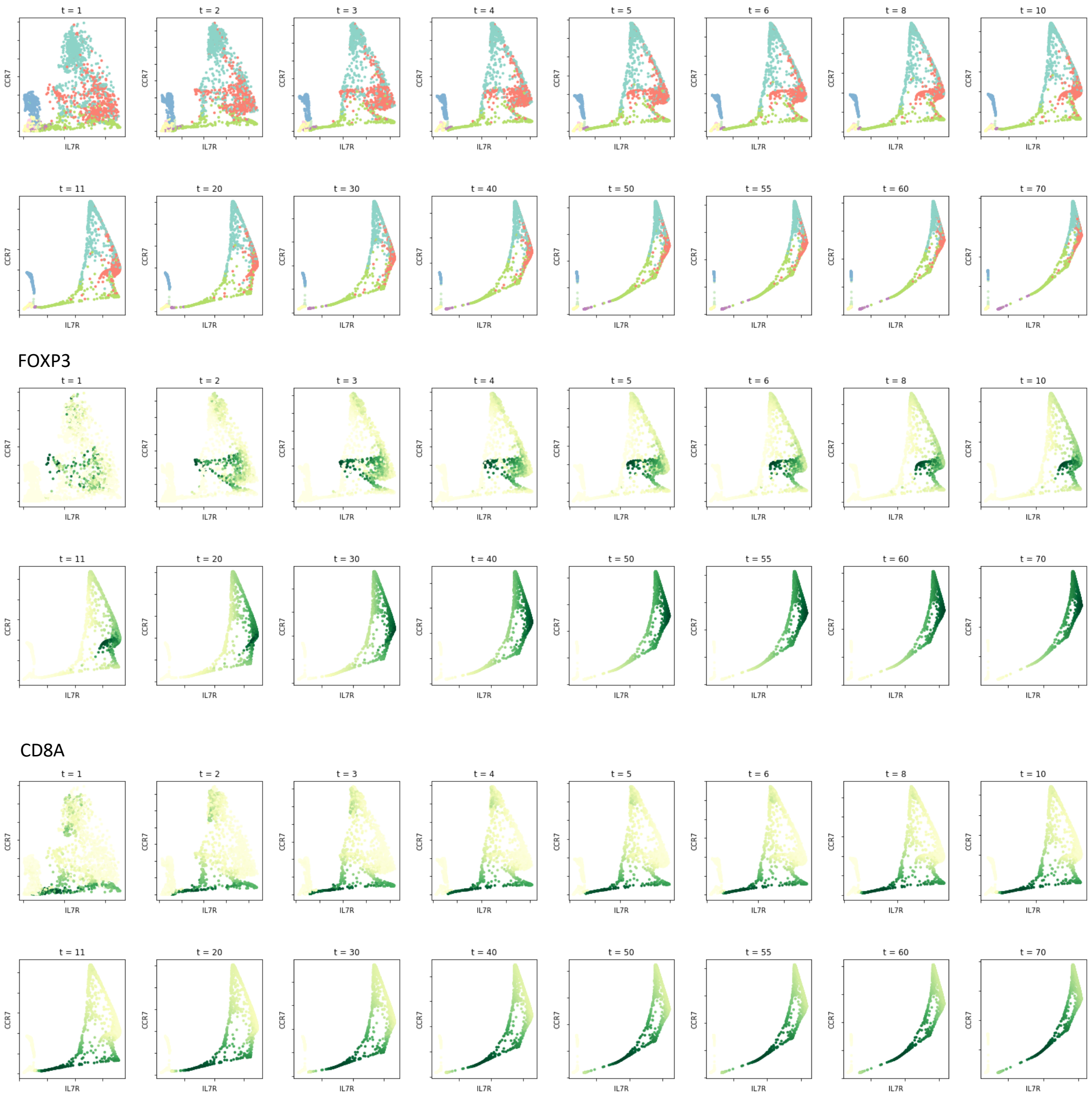

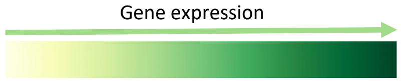

(E) Imputation evaluation of increasing values of t with knn=35 (more nearest neighbors) pca= 30 using MAGIC and sc-PHENIX(PCA-UMAP space). Here, the CCR7-IL7R interaction visualizing the PBMC clusters, recovered gene expression of FOXP3 and CD8A.

MAGIC imputation evaluation of pca (principal component dimension) with knn= 5 t= 5

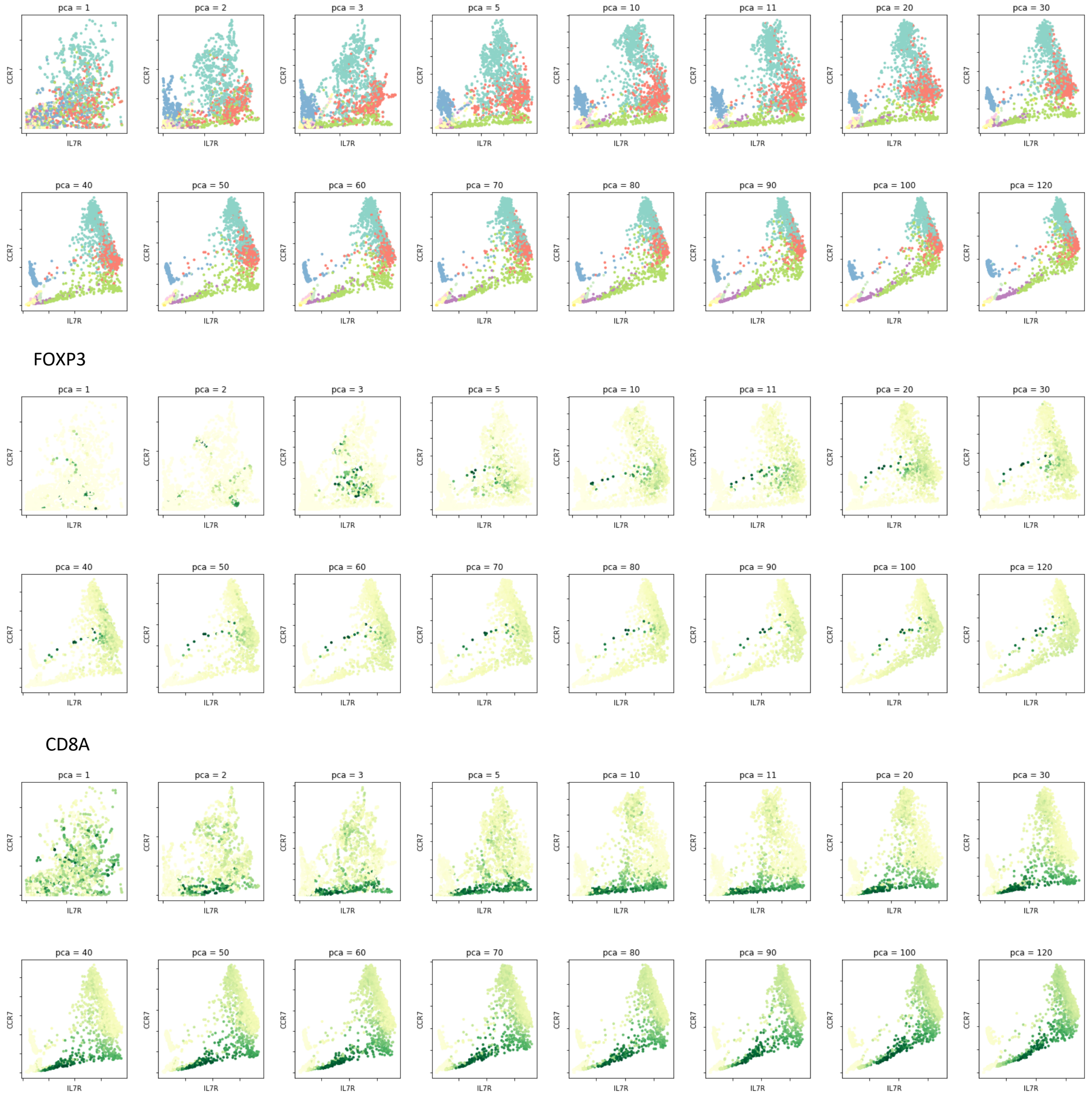

sc-PHENIX imputation evaluation of pca (principal component dimension) with knn= 5 t= 5

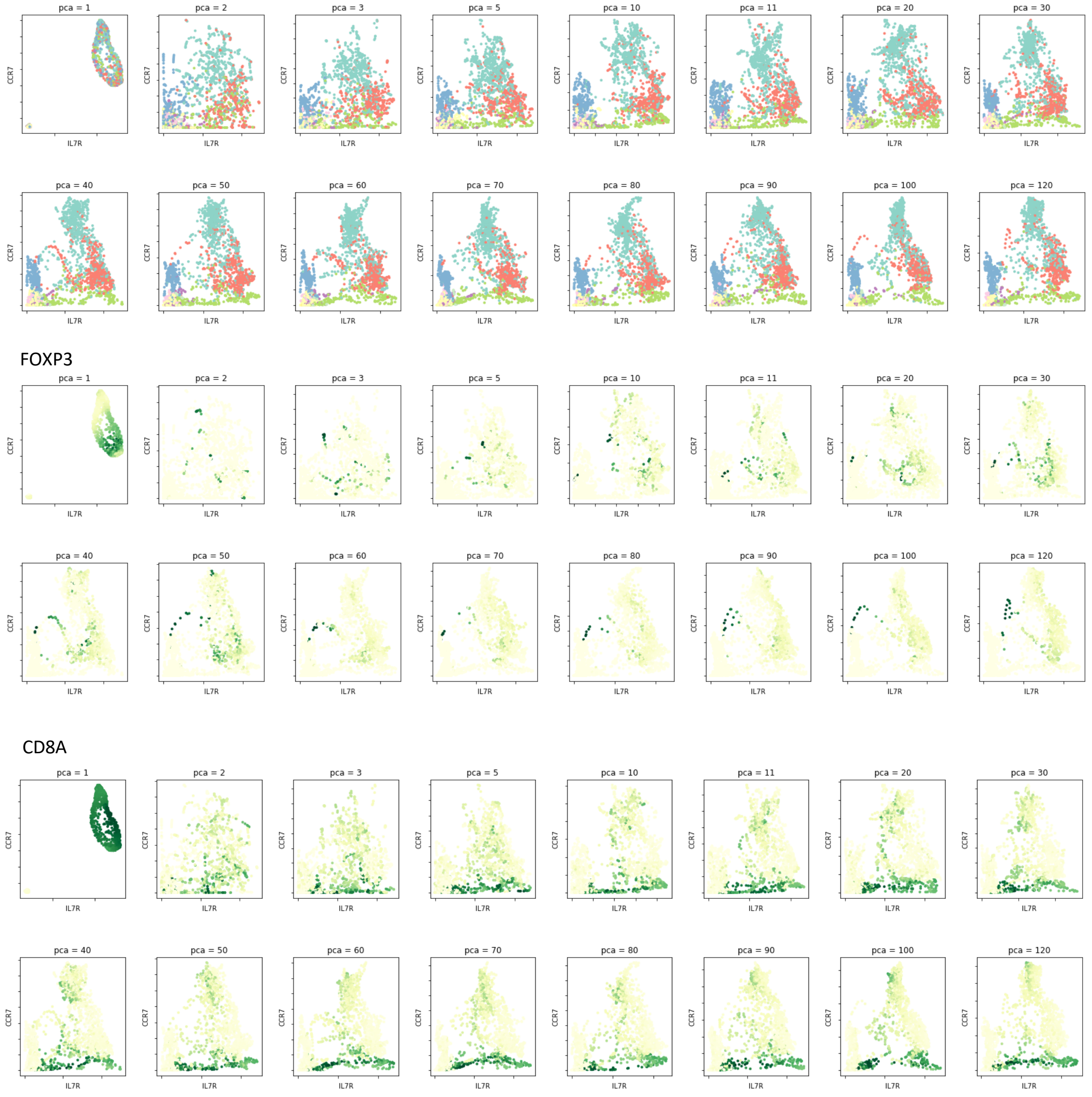

(F) Imputation evaluation of increasing values of principal components with knn=5 and t=5(less nearest neighbors and random walks) using MAGIC and sc-PHENIX(PCA-UMAP space). Here, the CCR7-IL7R interaction visualizing the PBMC clusters, recovered gene expression of FOXP3 and CD8A.

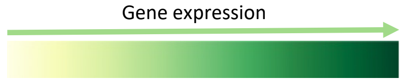

MAGIC imputation evaluation of pca (principal component dimension) with knn= 35 t= 10

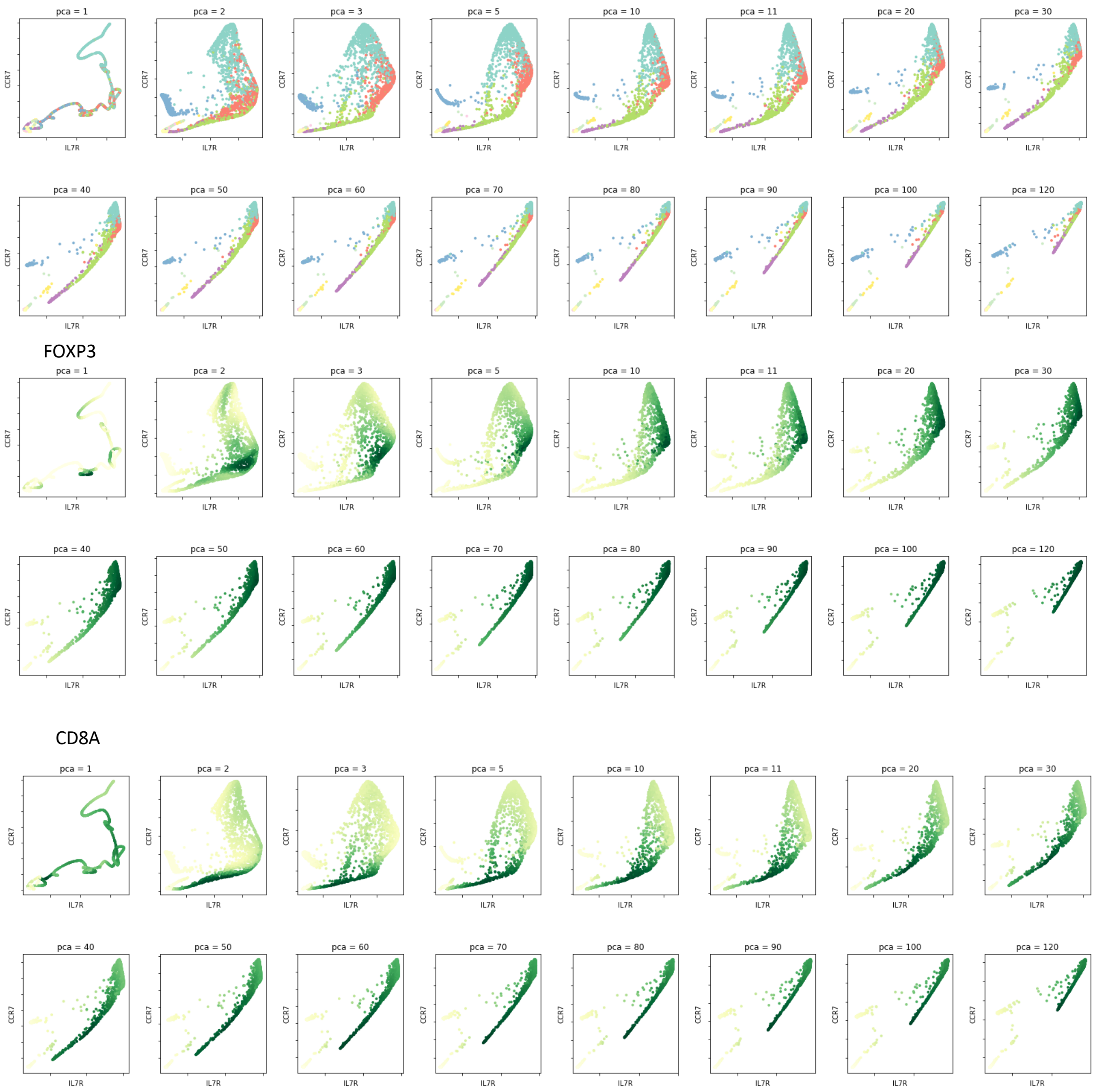

sc-PHENIX imputation evaluation of pca (principal component dimension) with knn= 35 t=10

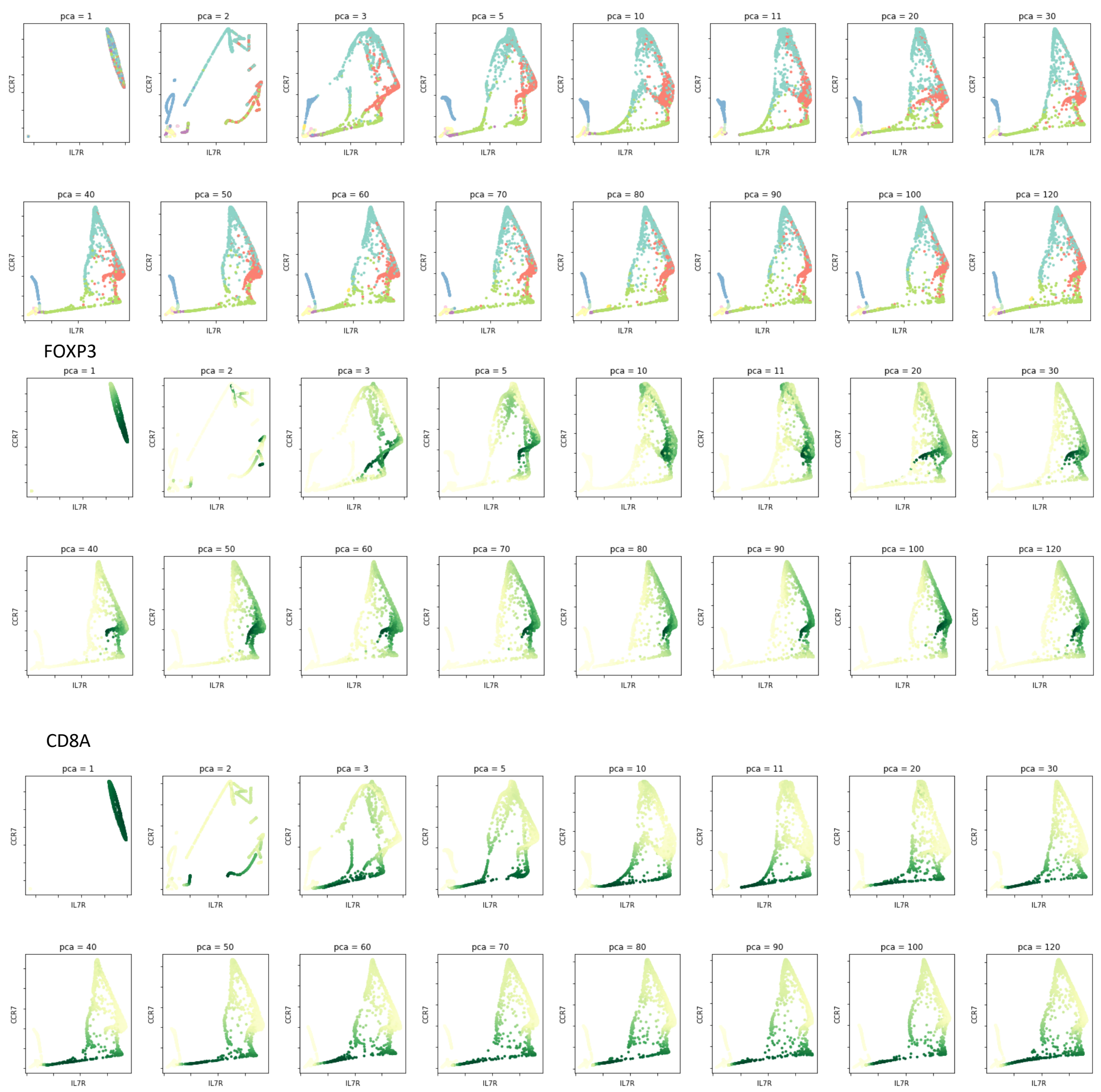

(G) Imputation evaluation of increasing values of principal components with knn=35 and t=10(more nearest neighbors and random walks) using MAGIC and sc-PHENIX(PCA-UMAP space). Here, the CCR7-IL7R interaction visualizing the PBMC clusters, recovered gene expression of FOXP3 and CD8A.

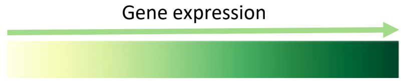

MAGIC imputation evaluation of pca (principal component dimension) with knn= 35 t= 20

sc-PHENIX imputation evaluation of pca (principal component dimension) with knn= 35 t=20

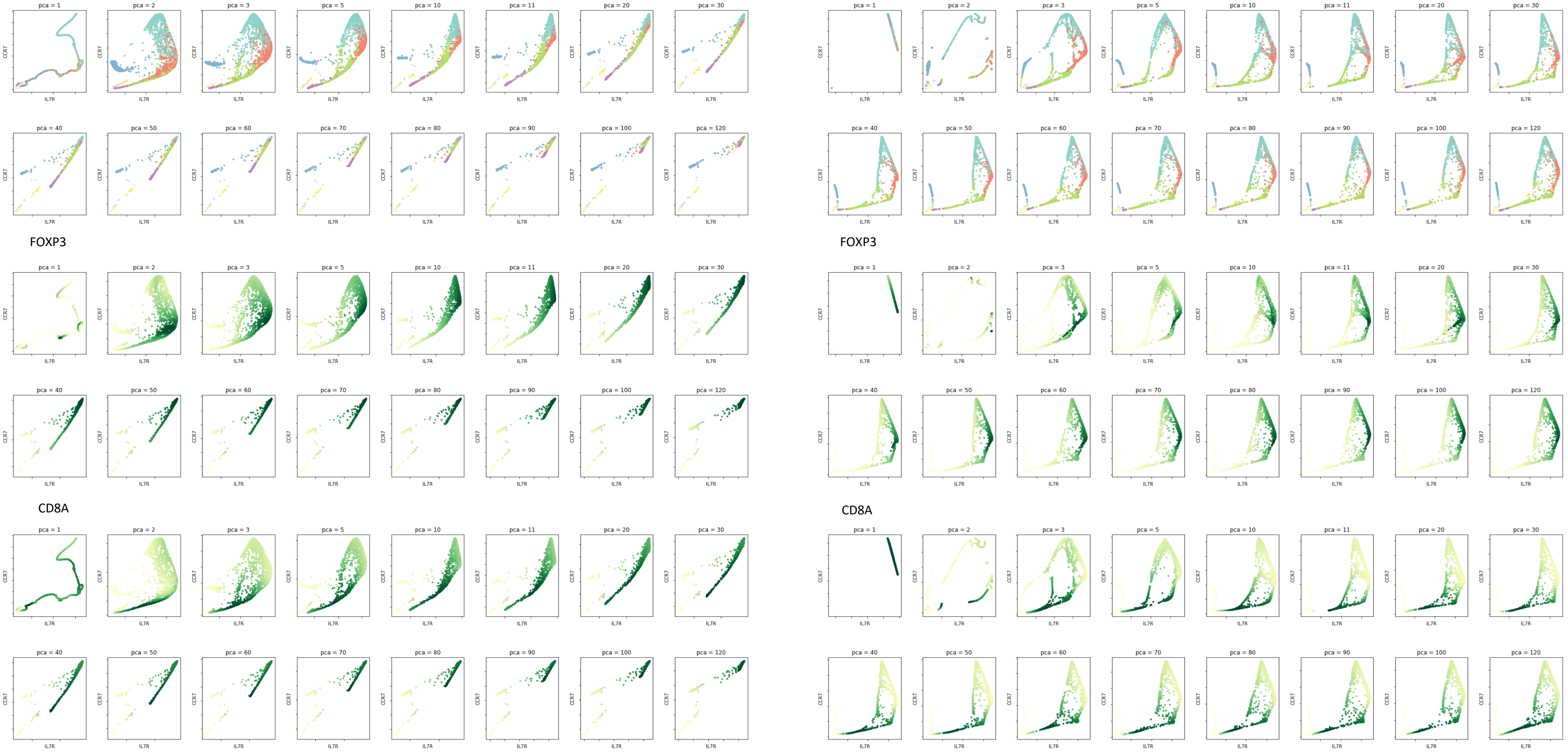

(H) Imputation evaluation of increasing values of principal components with knn=35 and t=20 (more nearest neighbors and much more random walks) using MAGIC and sc-PHENIX(PCA-UMAP space). Here, the CCR7-IL7R interaction visualizing the PBMC clusters, recovered gene expression of FOXP3 and CD8A.

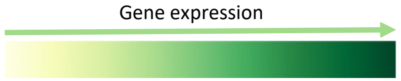

sc-PHENIX imputation evaluation of knn, without pca (k nearest-neighbors)with t= 5

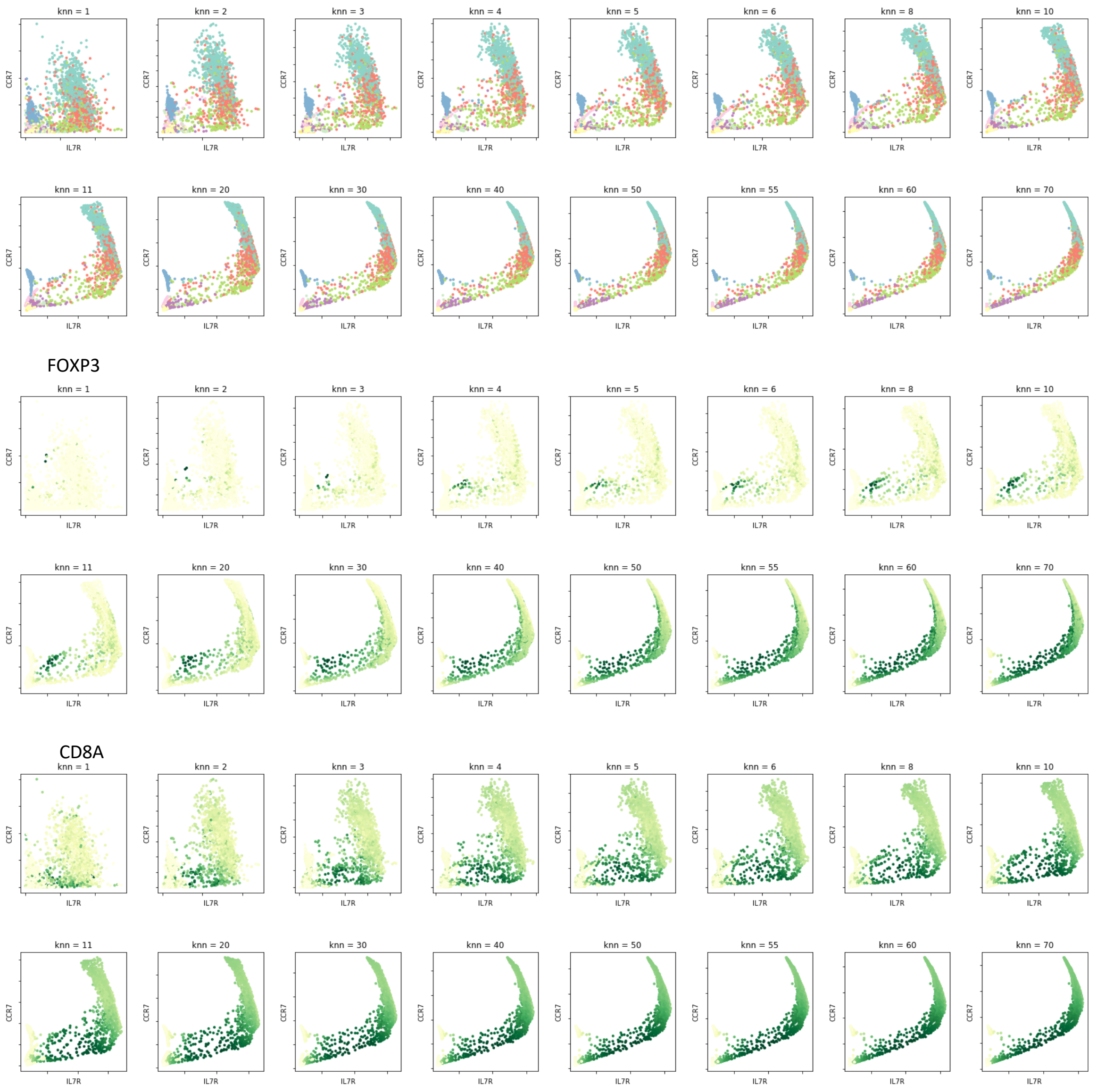

sc-PHENIX imputation evaluation of knn, without pca (k nearest-neighbors) with t= 10

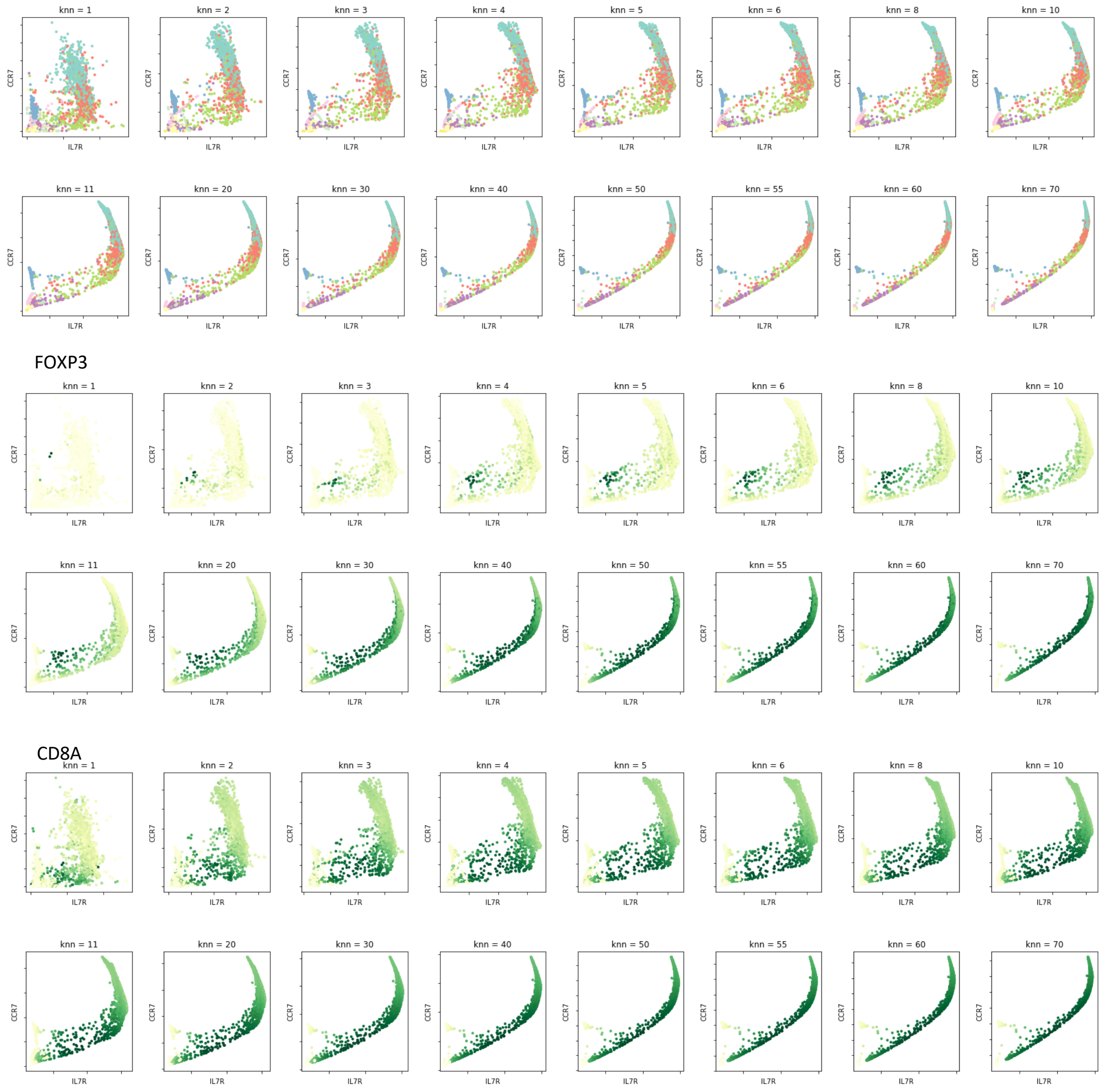

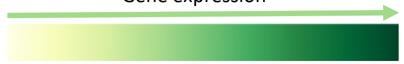

(I) Imputation evaluation of increasing values of knn with t=5 and t=10 (right and left respectively)using sc-PHENIX(only UMAP space). Here, the CCR7-IL7R interaction visualizing the PBMC clusters, recovered gene expression of FOXP3 and CD8A.

sc-PHENIX imputation evaluation of t (diffusion time), without pca with knn= 5

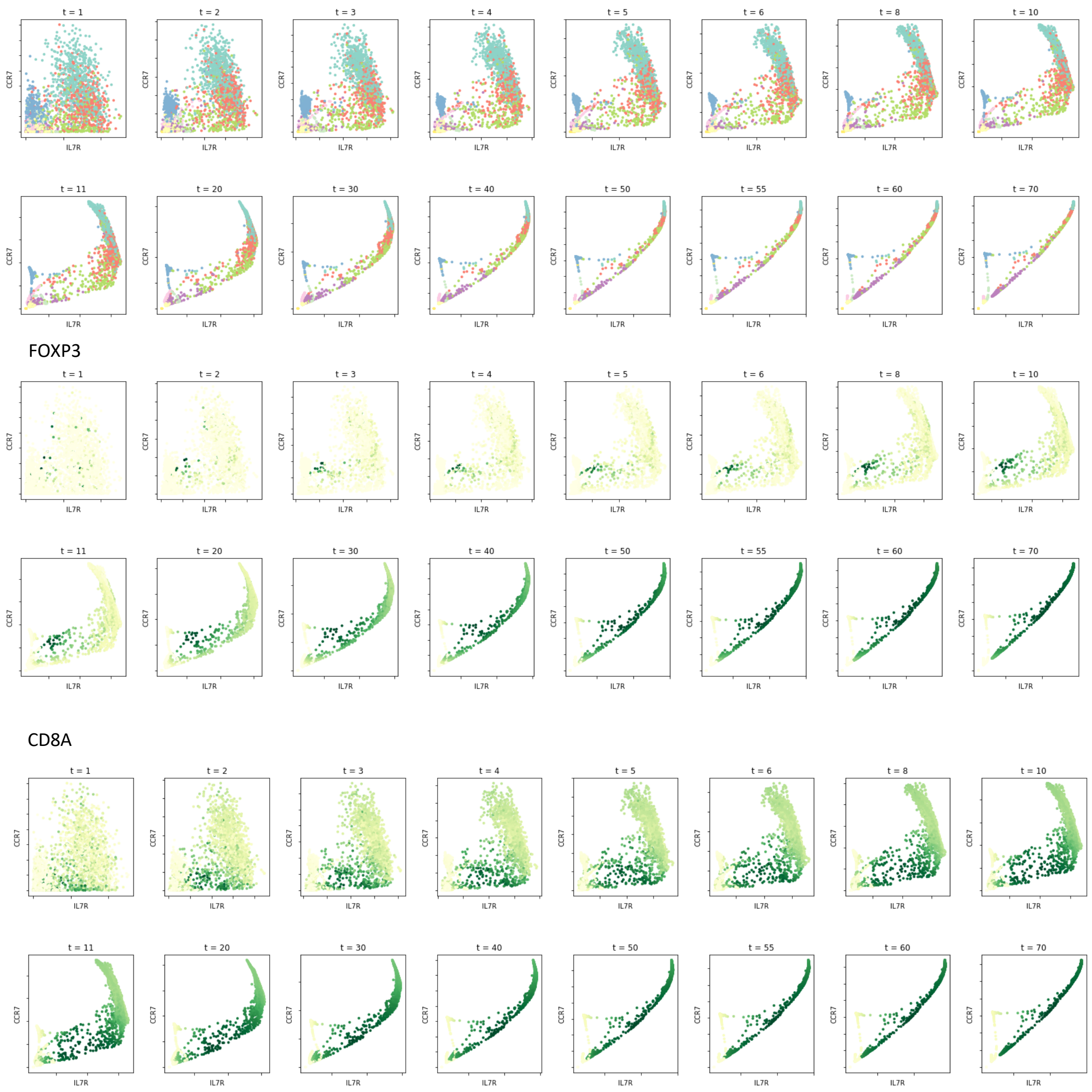

sc-PHENIX imputation evaluation of t (diffusion time) , without pca with knn= 35

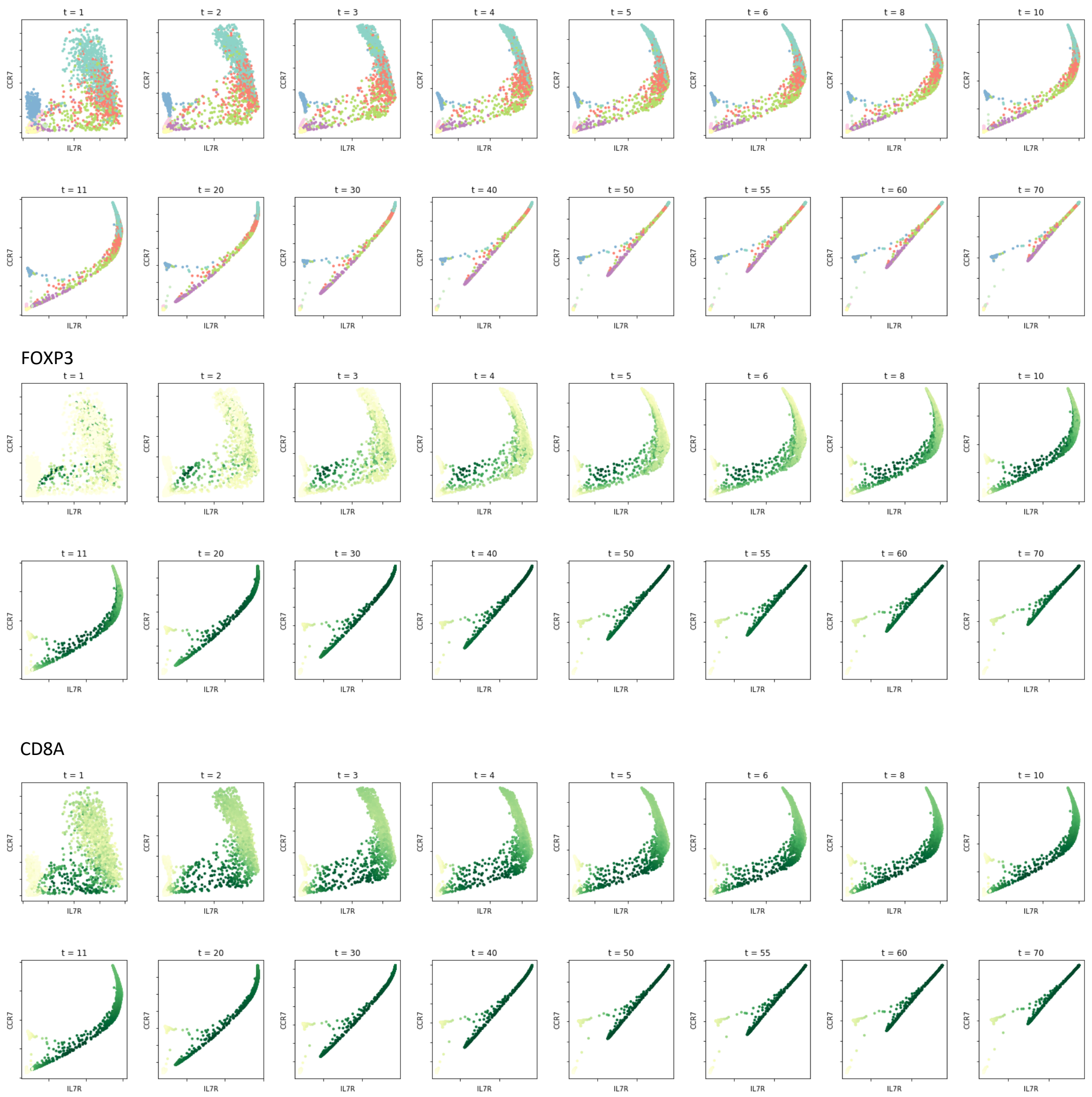

(I) Imputation evaluation of increasing values of t with knn=5 and knn=35 (right and left respectively) using sc-PHENIX (only UMAP space). Here, the CCR7-IL7R interaction visualizing the PBMC clusters, recovered gene expression of FOXP3 and CD8A.

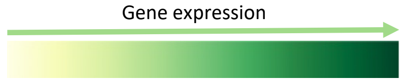

Supplement: Supplementary file 1 [file biology-13-00512-s001.zip › SM/Supple_ Sections/Section S4 pbmc gene gene interactions many parameters combinations.pdf]
