# Supplementary material for: Diffusion on PCA-UMAP Manifold: The Impact of Data Structure Preservation to Denoise High-Dimensional Single-Cell RNA Sequencing Data
Source: Biology (Basel). 2024 Jul 9;13(7):512. doi: 10.3390/biology13070512 (PMC11274112; doi:10.3390/biology13070512)
Supplement: Supplementary file 1 [file biology-13-00512-s001.zip › SM/Supple_ Methods/Methods S1.pptx]

## Slide 1
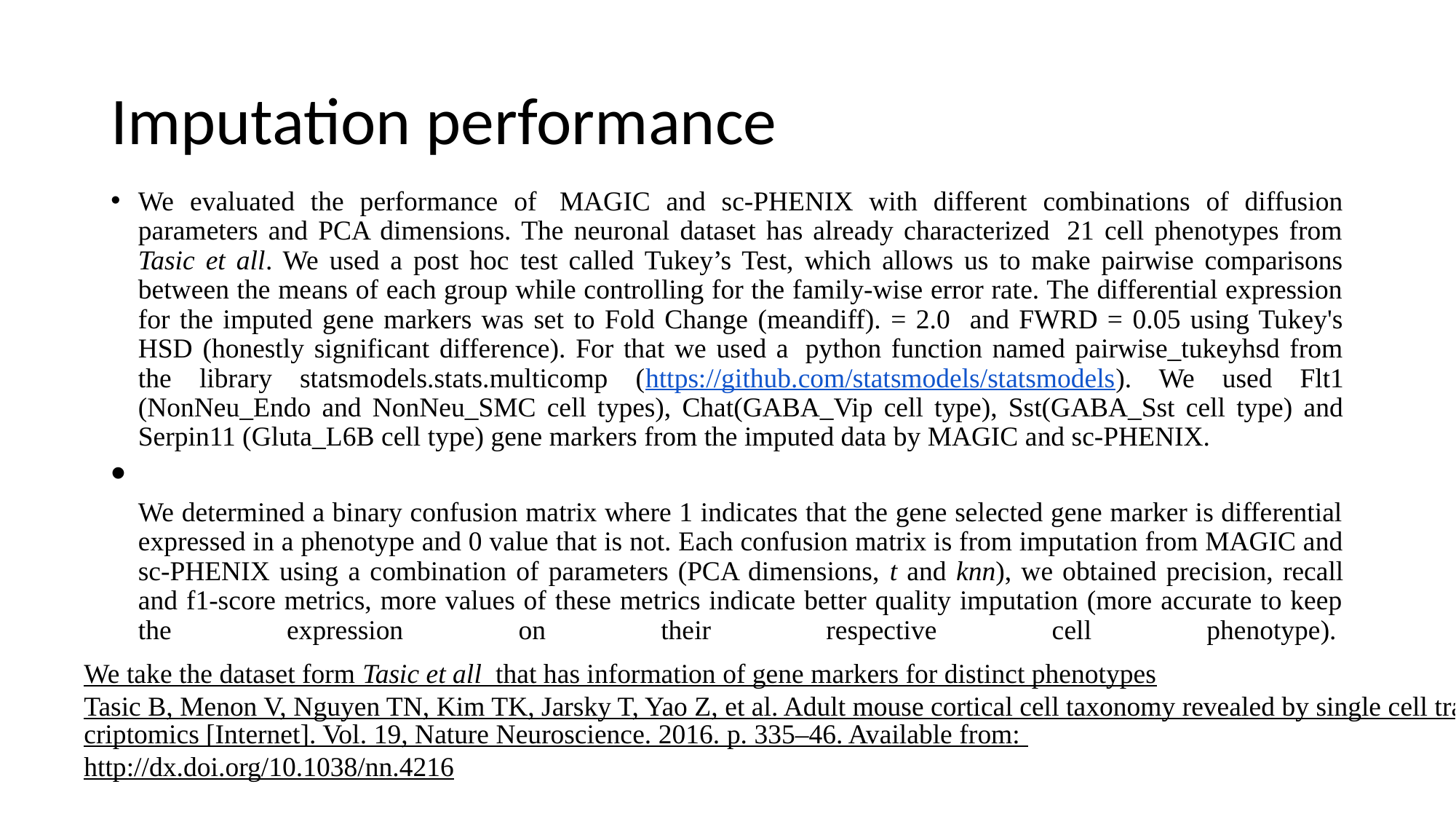

# Imputation performance
We evaluated the performance of  MAGIC and sc-PHENIX with different combinations of diffusion parameters and PCA dimensions. The neuronal dataset has already characterized  21 cell phenotypes from Tasic et all. We used a post hoc test called Tukey’s Test, which allows us to make pairwise comparisons between the means of each group while controlling for the family-wise error rate. The differential expression for the imputed gene markers was set to Fold Change (meandiff). = 2.0 and FWRD = 0.05 using Tukey's HSD (honestly significant difference). For that we used a  python function named pairwise_tukeyhsd from the library statsmodels.stats.multicomp (https://github.com/statsmodels/statsmodels). We used Flt1 (NonNeu_Endo and NonNeu_SMC cell types), Chat(GABA_Vip cell type), Sst(GABA_Sst cell type) and Serpin11 (Gluta_L6B cell type) gene markers from the imputed data by MAGIC and sc-PHENIX.
We determined a binary confusion matrix where 1 indicates that the gene selected gene marker is differential expressed in a phenotype and 0 value that is not. Each confusion matrix is from imputation from MAGIC and sc-PHENIX using a combination of parameters (PCA dimensions, t and knn), we obtained precision, recall and f1-score metrics, more values of these metrics indicate better quality imputation (more accurate to keep the expression on their respective cell phenotype).
We take the dataset form Tasic et all that has information of gene markers for distinct phenotypes
Tasic B, Menon V, Nguyen TN, Kim TK, Jarsky T, Yao Z, et al. Adult mouse cortical cell taxonomy revealed by single cell transcriptomics [Internet]. Vol. 19, Nature Neuroscience. 2016. p. 335–46. Available from: http://dx.doi.org/10.1038/nn.4216

## Slide 2
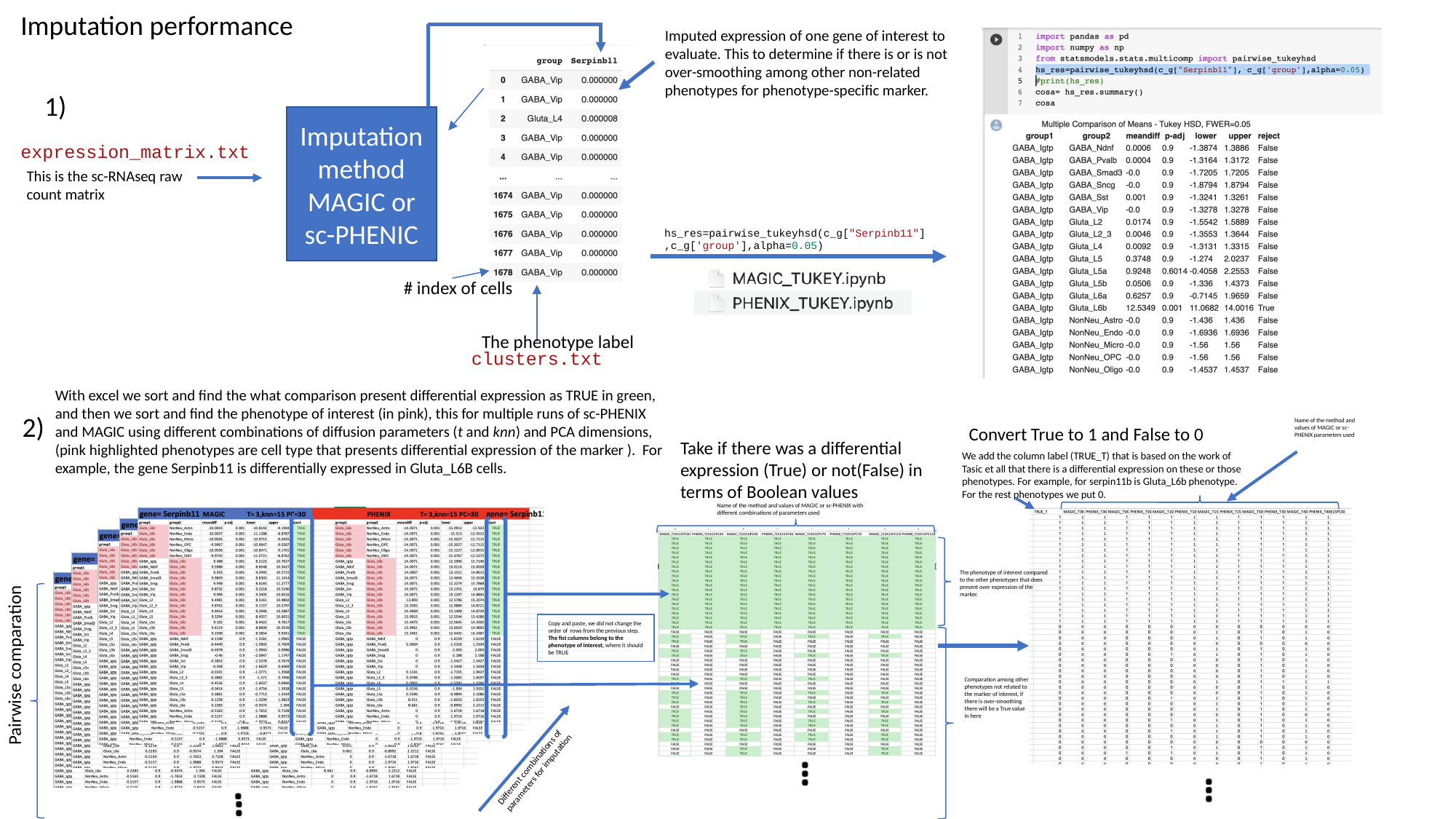

Imputation performance
Imputed expression of one gene of interest to evaluate. This to determine if there is or is not over-smoothing among other non-related phenotypes for phenotype-specific marker.
1)
Imputation method
MAGIC or sc-PHENIC
expression_matrix.txt
This is the sc-RNAseq raw count matrix
hs_res=pairwise_tukeyhsd(c_g["Serpinb11"] ,c_g['group'],alpha=0.05)
# index of cells
The phenotype label
clusters.txt
With excel we sort and find the what comparison present differential expression as TRUE in green, and then we sort and find the phenotype of interest (in pink), this for multiple runs of sc-PHENIX and MAGIC using different combinations of diffusion parameters (t and knn) and PCA dimensions, (pink highlighted phenotypes are cell type that presents differential expression of the marker ). For example, the gene Serpinb11 is differentially expressed in Gluta_L6B cells.
2)
Name of the method and values of MAGIC or sc-PHENIX parameters used
Convert True to 1 and False to 0
Take if there was a differential expression (True) or not(False) in terms of Boolean values
We add the column label (TRUE_T) that is based on the work of Tasic et all that there is a differential expression on these or those phenotypes. For example, for serpin11b is Gluta_L6b phenotype. For the rest phenotypes we put 0.
Name of the method and values of MAGIC or sc-PHENIX with different combinations of parameters used
The phenotype of interest compared to the other phenotypes that does present over expression of the marker.
Copy and paste, we did not change the order of rows from the previous step. The fist columns belong to the phenotype of interest, where it should be TRUE
Pairwise comparation
Comparation among other phenotypes not related to the marker of interest, if there is over-smoothing there will be a True value in here
Different combinations of parameters for imputation

## Slide 3
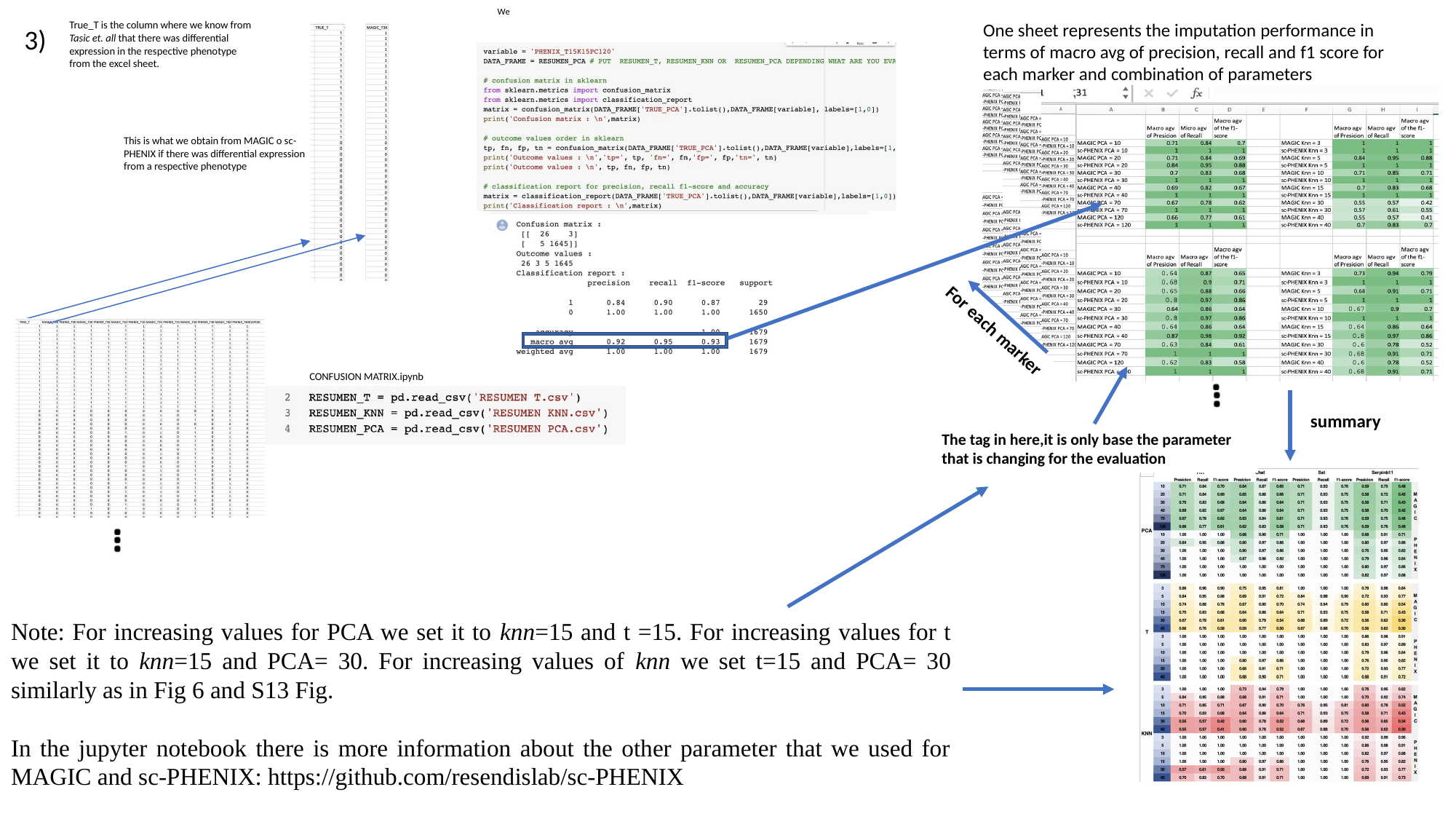

We
True_T is the column where we know from Tasic et. all that there was differential expression in the respective phenotype from the excel sheet.
One sheet represents the imputation performance in terms of macro avg of precision, recall and f1 score for each marker and combination of parameters
3)
This is what we obtain from MAGIC o sc-PHENIX if there was differential expression from a respective phenotype
For each marker
CONFUSION MATRIX.ipynb
summary
The tag in here,it is only base the parameter that is changing for the evaluation
Note: For increasing values for PCA we set it to knn=15 and t =15. For increasing values for t we set it to knn=15 and PCA= 30. For increasing values of knn we set t=15 and PCA= 30 similarly as in Fig 6 and S13 Fig.
In the jupyter notebook there is more information about the other parameter that we used for MAGIC and sc-PHENIX: https://github.com/resendislab/sc-PHENIX
