# Supplementary material for: Diffusion on PCA-UMAP Manifold: The Impact of Data Structure Preservation to Denoise High-Dimensional Single-Cell RNA Sequencing Data
Source: Biology (Basel). 2024 Jul 9;13(7):512. doi: 10.3390/biology13070512 (PMC11274112; doi:10.3390/biology13070512)
Supplement: Supplementary file 1 [file biology-13-00512-s001.zip › SM/Supple_ Sections/Section S1 sc-PHENIX as MAGIC reproducibility.pdf]

Here we show that our diffusion process with sc-PHENIX (with PCA initialization) is similar to MAGIC. The imputed gen-gen interactions have the same structure (Fig A top). With sc-PHENIX (PCA-initialization) and MAGIC (Fig I bottom) the R2 correlations are near 1. This indicates that the imputation from both approaches are similar. Therefore, in an indirect manner, we can determine that the exponentiated Markov Matrix of sc-PHENIX is computed as MAGIC when the input is PCA. Data is from EMT data from MAGIC's paper.

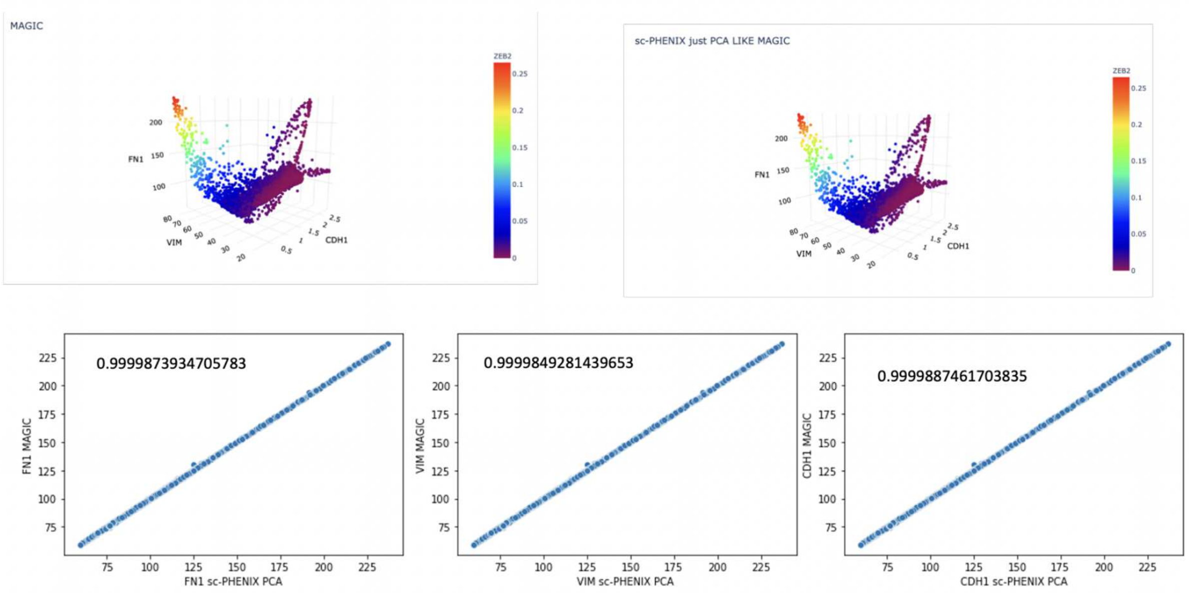

Fig A. sc-PHENIX with PCA initialization reproducibility as MAGIC

Top: VIM-FN1-CDH1 interaction from MAGIC and sc-PHENIX with PCA initialization. Bottom: Scatter plot of the imputed expression of FN1(left), VIM (middle) and CDH1(right) from MAGIC and sc-PHENIX with PCA initialization.

Additionally, given PCA's robustness and the algebraic nature of creating diffusion maps, we anticipated similar outcomes in theory. However, in light of your feedback, we realize our approach might still need refinement due to the libraries used for matrix operations in MAGIC (Fig B). To assess the reproducibility of sc-PHENIX with PCA initialization against MAGIC, we computed the mean R2 and its standard deviation from the gene-to-gene correlations in both imputed matrices. The mean R2 was approximately 0.999, with a very small standard deviation, indicating that results from sc-PHENIX with PCA initialization might closely resemble those from MAGIC. Nevertheless, with 28,910 gene symbols in this dataset, it was critical to determine if significant outliers were obscured by the data's sheer volume. We plotted gene values from MAGIC against those from sc-PHENIX (init PCA) for R^2 values below 0.998, identifying only seven genes under this threshold. These findings are presented in the supplementary section and suggest our method replicates MAGIC's results well. A possible explanation for any minor discrepancies might be MAGIC's use of a library for sparse data, which is merely a hypothesis.

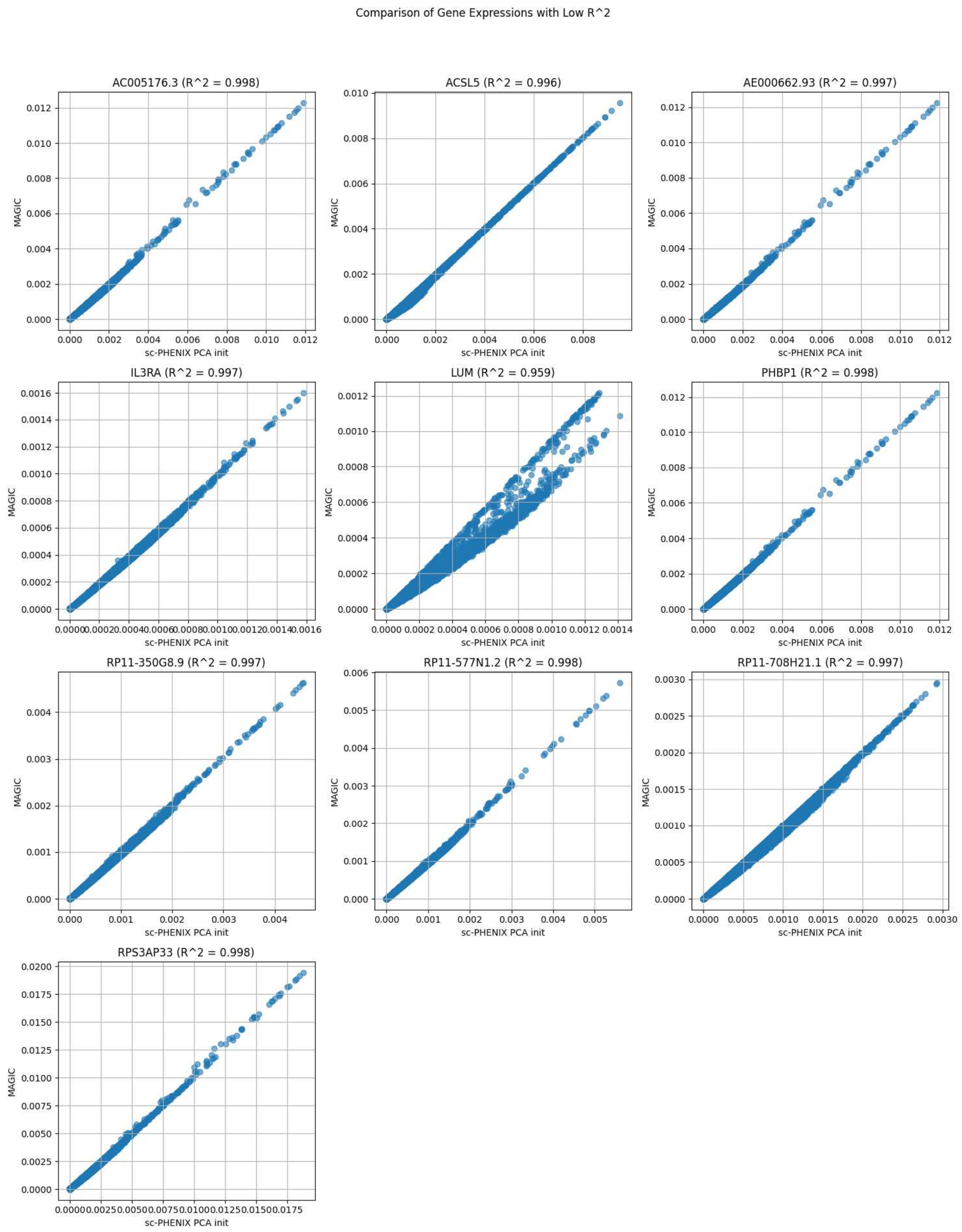

Figure B Comparison of Gene Expressions with Low R^2 Correlation between MAGIC and sc-PHENIX with PCA Initialization

Graphical representation of the correlation between imputed gene values in the EMT dataset by sc-PHENIX with PCA initialization and MAGIC. Genes with R^2 values below 0.998 are highlighted to focus on those with the greatest discrepancies, indicating only seven genes significantly different. The correlation between gene values was calculated using linear regression, and genes with a determination coefficient R^2 below 0.998 were plotted.

This analysis underscores the high reproducibility of the methods employed and suggests the possibility of minor computational differences related to the use of libraries.
