# Supplementary material for: Diffusion on PCA-UMAP Manifold: The Impact of Data Structure Preservation to Denoise High-Dimensional Single-Cell RNA Sequencing Data
Source: Biology (Basel). 2024 Jul 9;13(7):512. doi: 10.3390/biology13070512 (PMC11274112; doi:10.3390/biology13070512)
Supplement: Supplementary file 1 [file biology-13-00512-s001.zip › SM/Supple_ Sections/Section S11 Other aplications of sc-PHENIX.pdf]

sc-PHENIX uses UMAP, therefore can use its different variants that can be used for other types of data such as microbiome data (superviced UMAP space) and data integration of two technologies such CITE-seq (UMAP model combination). Supervised UMAP (embeddings for highly heterogeneous data), non-parametric UMAP (embedding the connectivity matrix with neural networks), combination of UMAP models (embedding integrating different datasets), Mutual k-NN Graph (Improving the Separation Between Similar Classes) and DensMAP (Better Preserving Local Density). For example, UMAP can combine two topologies from two omics technologies dataset such as CITE-seq (single-cell transcriptomics and proteomics profiles in one experiment). Then having combined these two topologies in an embedding, sc-PHENIX can use it to impute the single-cell-transcriptomic matrix or the proteomic. Another possible implementation is the high-heterogeneous microbiota data from different technologies (16S rRNA and shotgun Whole Metagenome Sequence), the microbiota matrix contains many zeros and it drives distortion of the microbe abundance distributions and few differential microbes among studie cases. It is impossible to obtain a well-cluster structure among samples (sparsity aggravates this), especially in clinical data among patients. For this, the manifold of the supervised UMAP embedding leads to sc-PHENIX imputation to obtain more differential microbes among studie cases, for more details we present mb-PHENIX [1] , a superviced version of sc-PHENIX for microbiota data. Also SHAP values[2]a popular approach to reveal important taxon involved in disease [3]. The SHAP value matrix can be transformed with diffusion on UMAP space to denoised microbiota data.

Thus, UMAP variants can aid the limitations of UMAP for different contexts. For example, the initial k nearest neighbor (k-NN) graph, which connects each datapoint to its k nearest neighbors based on some distance metric, is constructed and used to generate the initial topological representation of a dataset. However, previous research has shown that using the initial k-NN graph of UMAP may not provide an accurate representation of the underlying local structure for a high dimensional dataset [4]. Thus, it is proposed Mutual k-NN graphs combined with UMAP (thus a UMAP variant) have been shown to contain many desirable properties when combating the "curse of dimensionality", this is reflected in better conservation of local structure [4]. However, the implementation of the mutual k-NN graphs combined with UMAP is not user friendly at the time.

Another strategy to preserve more local structure is to re-run a UMAP subsection, something used in a "re-clustering" analysis [5]; fewer samples facilitates embedding to preserve local structure better. This strategy has a higher resolution among a group of similar cells [6], at least in visual projections of UMAP plots. However, the imputation based on a subsection of cells does not contemplate the other distinct cell phenotypes, making it inadequate to compare imputed expression cells outside the subsection. We acknowledge for sc-PHENIX, the previouslyprevious mentioned has detrimental effects oin local structure for imputation, but it is mitigated by re-clustering of sub sections.

1. Padron-Manrique C, Vázquez-Jiménez A, Esquivel-Hernandez DA, Lopez YEM, Neri-Rosario D, Sánchez-Castañeda JP, et al. mb-PHENIX: Diffusion and Supervised Uniform Manifold Approximation for denoising microbiota data. bioRxiv. 2022. p. 2022.06.23.497285. doi:10.1101/2022.06.23.497285

2. Lundberg SM, Lee S-I. A Unified Approach to Interpreting Model Predictions. Adv Neural Inf Process Syst. 2017;30. Available: https://proceedings.neurips.cc/paper/2017/file/8a20a8621978632d76c43dfd28b67767-Paper.pdf

3. Gou W, Ling C-W, He Y, Jiang Z, Fu Y, Xu F, et al. Interpretable Machine Learning Framework Reveals Robust Gut Microbiome Features Associated With Type 2 Diabetes. Diabetes Care. 2021;44: 358.

4. Dalmia A, Sia S. Clustering with UMAP: Why and How Connectivity Matters. 2021 [cited 30 Jul 2022]. doi:10.48550/arXiv.2108.05525

5. Packer JS, Zhu Q, Huynh C, Sivaramakrishnan P, Preston E, Dueck H, et al. A lineage-resolved molecular atlas of C. elegans embryogenesis at single cell resolution. bioRxiv. bioRxiv; 2019. doi:10.1101/565549

6. Khodadadi-Jamayran A, Tsirigos A. Graph drawing-based dimensionality reduction to identify hidden communities in single-cell sequencing spatial representation. bioRxiv. bioRxiv; 2020. doi:10.1101/2020.05.05.078550
