# Supplementary material for: Diffusion on PCA-UMAP Manifold: The Impact of Data Structure Preservation to Denoise High-Dimensional Single-Cell RNA Sequencing Data
Source: Biology (Basel). 2024 Jul 9;13(7):512. doi: 10.3390/biology13070512 (PMC11274112; doi:10.3390/biology13070512)
Supplement: Supplementary file 1 [file biology-13-00512-s001.zip › SM/Supple_ Sections/Section S2 Laplacian Eigenmaps initialization of UMAP _.pdf]

When using Laplacian Eigenmaps for UMAP as the embedding initialization for sc-PHENIX with high values of n_components (fixed at 190), the metrics—Pearson Correlation, Spearman Score, and R2 Score—show a rapid increase up to around 20 knn, after which they stabilize. The area under the curve (AUC) for these metrics are 0.68 for Pearson Correlation, 0.69 for Spearman Score, and 0.51 for R2 Score, indicating strong performance. This suggests that for higher dimensional initialization, an optimal knn value is around 20 for achieving better performance in terms of these metrics.

Similarly, when using low values of n_components (fixed at 20), the metrics also show improvement and stabilization with increasing knn, though with less pronounced fluctuations compared to the high n_components scenario. The AUC for these metrics in the low n_components scenario are 0.68 for Pearson Correlation, 0.69 for Spearman Score, and 0.50 for R2 Score, showing consistent performance. This suggests that for lower dimensional initialization, the performance stabilizes quickly with an optimal knn value also around 20.

In both cases, the metrics demonstrate significant improvement and stabilization around a knn value of 20, with consistent AUC values, highlighting this as a critical point for optimizing the initialization parameters for sc-PHENIX using Laplacian Eigenmaps. However, the use of Laplacian Eigenmaps does not super pass PCA-UMAP initialization for sc-PHENIX (see main text, Fig 2)

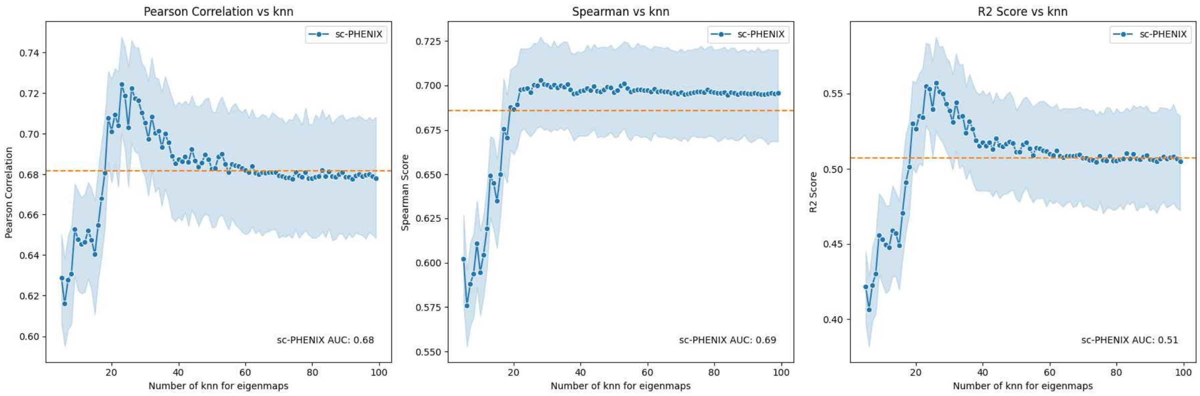

Figure A: Use of Laplacian Eigenmaps with UMAP for sc-PHENIX Initialization with High Values of n_components

Laplacian Eigenmaps for UMAP are used as the embedding initialization for sc-PHENIX, with n_components fixed at 190 while varying the values of knn. Significant improvements and stabilization are observed around a knn value of 20. The dotted line is the global mean of all samples for its metric.

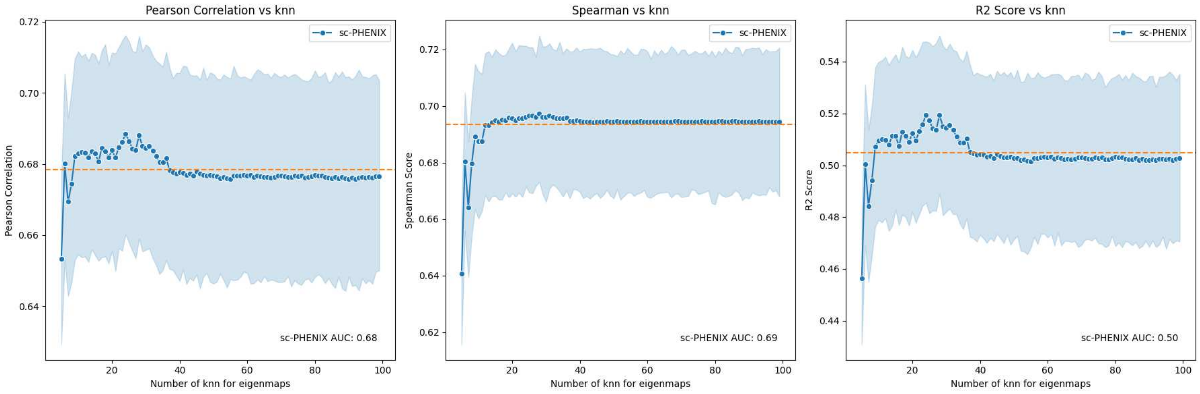

Figure B: Use of Laplacian Eigenmaps with UMAP for sc-PHENIX Initialization with Low Values of n_components

Laplacian Eigenmaps for UMAP are used as the embedding initialization for sc-PHENIX, with n_components fixed at 20 while varying the values of knn. Significant improvements and stabilization are observed around a knn value of 20.
