# Supplementary material for: Diffusion on PCA-UMAP Manifold: The Impact of Data Structure Preservation to Denoise High-Dimensional Single-Cell RNA Sequencing Data
Source: Biology (Basel). 2024 Jul 9;13(7):512. doi: 10.3390/biology13070512 (PMC11274112; doi:10.3390/biology13070512)
Supplement: Supplementary file 1 [file biology-13-00512-s001.zip › SM/Supple_ Sections/Section S3 pbmc gene gene interactions.pdf]

Evaluation of Peripheral Blood Mononuclear Cells Dataset

To continue with the sc-PHENIX evaluation and test their applicability, we used the dataset of 3k Peripheral Blood Mononuclear Cells (PBMC) freely available from 10X Genomics. This dataset has different cell phenotypes (Fig I).

We evaluated the effect of over-smoothing on two gene-gene interactions recovered by sc-PHENIX, MAGIC, and SAVER. First, we used the IL7R-CCR7 interaction. The IL7R-CCR7 interaction is related to the transition dynamics of a nävie to a memory CD4+ T-cell state. This transition involves a **CCR7** downregulation [1]. Also, **IL7R** is expressed in both nävie CD4+ and memory CD4+ T cell types but stimulates the proliferation of mature CD4+ T cells [1].

The second gene-gene interaction is CCR7-PTPRC. The expression of CD45RA (also known as **PTPRC**) is generally associated with näive T cells. However, a subset of $CD45RA^{+}CCR7^{-}$effector memory T cells re-expresses CD45RA in cytometry experiments [3]. It is interesting to evaluate the recovered expression of the CCR7-ILR7 and CCR7-PTPRC interactions by the scRNA-seq imputation methods. For the non-imputed data (Fig IA), it is infrequent to find strong relationships when the data present high rates of dropouts, especially a relationship that explains the transition dynamics of cell states [2]. Therefore in the non-imputed data, the IL7R-CCR7 or PTPRC-IL7R interactions are only two examples of a gene-gene interaction misrepresentation. However, MAGIC, sc-PHENIX, and SAVER recover IL7R-CCR7 and CCR7-PTPRC interaction, recreating the biaxial plots typically in flow cytometry. The SAVER (model based imputation method) seems to recover some relationship of the gene-gene interactions but it has no well-defined cluster structure (Fig IA). Also, there is no present continuum structure showing the transition of naive memory cells. SAVER fails to obtain the gene dynamics of the maturation of the proliferation of mature CD4+ or the $CD45RA^{+}CCR7^{-}$effector memory T cells that re-expresses $CD45RA^{+}$.

MAGIC and sc-PHENIX share the same diffusion process (smoothing based methods). Therefore, *knn* and *t* parameters are used in the same way in both methods, the main difference is that MAGIC uses PCA space to measure cell distances and in this section sc-PHENIX uses PCA-UMAP space instead. Moreover, as already mentioned, imputation carries over-smoothing in data. We evaluated the effect of over-smoothing, using distinct increasing values of *knn* and *t* for the IL7R-CCR7 interaction for both diffusion approaches (Fig IA and B and S2 Section). MAGIC recovers IL7R and CCR7 values in other cell phenotypes different from nävie CD4+ and memory CD4+ cell types. Thus, MAGIC over smoothed data (Fig IA, Fig II and, S2 Section).

Additionally, in S2 Section we evaluated ILR7-CCR7 interaction recovered by the MAGIC and sc-PHENIX with UMAP and PCA-UMAP space through increasing principal component dimensions combined with low and high values of diffusion parameters (*knn* and *t*). The sc-PHENIX (PCA-UMAP space) imputation maintains a IL7R-CCR7 interaction through different increasing values of diffusion parameters; over-smoothing has not a higher effect compared to MAGIC (S4 Section, Fig IA and B, Fig II).

However, MAGIC imputation using low values of diffusion parameters (*knn* and *t*), a more*"local imputation"*, recovers to some extent the transition dynamic of the nävie to a memory CD4+ cell state. But we can observe an over-smoothing of IL7R (in high levels) across some NK cells (Fig IB, knn=5 and t=5 with MAGIC), NK generally do not express IL7R [4]. With MAGIC, increasing values of diffusion parameters (*knn* and *t*), the dynamic transition is lost by over-smoothing (Fig IB and S2

Section). As a consequence, the continuum structure is also lost.

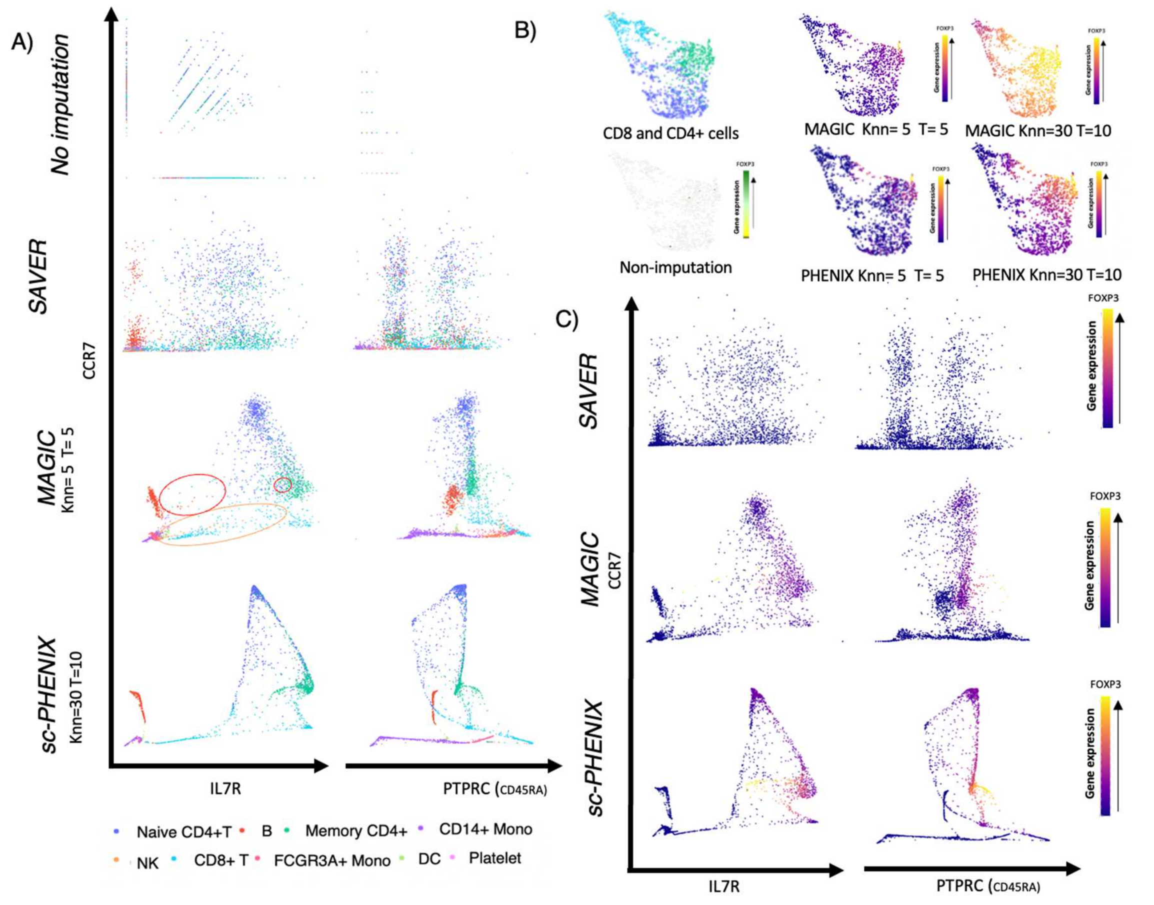

Fig I . Single-cell RNA-seq imputation algorithms apply to the 3k PBMC dataset.

A) IL7R-CCR7 interaction of the non-imputed 3k PBMC dataset and the imputed by MAGIC, SAVER, and sc-PHENIX. Distinct PBMC cell phenotypes are shown below. Red ovals indicate B cells overs-mooted with increasing values of CCR7 in B cells. Orange ovals indicate NK cells over-smoothed with increasing values of CCR7.

B) Sections of the UMAP plot of the 3k PBMC dataset showing the CD8+, memory CD4+ and näive CD4+ T cells to show the imputation values of FOXP3 by MAGIC and sc-PHENIX imputation (imputation methods using diffusion maps). The CD8+, memory CD4+ and näive CD4+ T cells clusters (above left). Non-imputed data values of FOXP3 on the UMAP projection (below left). FOXP3 recovered values by MAGIC shown on the UMAP projection, using different values of diffusion parameters of knn and t (above middle and right). FOXP3 recovered values by sc-PHENIX shown on the UMAP projection, using different values of diffusion parameters of knn and t (below middle and right). C) IL7R-CCR7 and PTPRC-CCR7 interactions of the recovered 3k PBMC dataset by MAGIC, SAVER and sc-PHENIX. Recovered values of FOXP3 are shown on the IL7R-CCR7 and PTPRC-CCR7 recovered interactions.

In both gene-gene interactions recovered by MAGIC, the local and continuum structure is not well captured as in sc-PHENIX using PCA-UMAP space as initialization. To avoid over-smoothing with MAGIC is to generally pick a *knn* such that it is the smallest value that still results in a connected graph[5]. Also mentions that it is robust to the number of PCA dimensions (10 to 100 PC's). However, we observed in S4 Section that MAGIC is not robust to the number of PCA dimensions nor to local imputation (low diffusion parameters knn and t). Because, the effect of over-smoothing grows as PCA dimensionality and diffusion parameters values increase. Generally, we observe that MAGIC severely distorts data by over-smoothing.

In S4 Section additionally to evaluate over-something with additional genes, we projected the gene expression of two genes (CD8A and FOXP3) on the IL7R-CCR7 interaction. With MAGIC the CD8A (a marker for CD8+ T cell), CD8A expression is over-smoothed in distinct cell phenotypes that are not

CD8+ T cells. Also, with MAGIC set to a local imputation (*knn* =5 and *t*=5) this subpopulation appears but as mentioned before, the data is over-smoothed in a local imputation (Fig 4C and S3). Therefore, even though MAGIC is set to a local imputation does not stop over-smooth the data.

With MAGIC we observe (Fig IB and C and S4 Section) the presence of the subpopulation of $CD45RA^{+}CCR7^{-}$effector memory T cells that re-expresses $CD45RA^{+}$. Also this population expresses more FOXP3. FOXP3 is a member of the forkhead transcription factor family. Unlike other members, it is mainly expressed in a subset of CD4+ T-cells that play a suppressive role in the immune system [3]. However, in fig IC or S4 Section, it is easier to detect this subpopulation sc-PHENIX (PCA-UMAP space) rather than MAGIC or sc-PHENIX (only UMAP space). With all combinations of diffusion parameters sc-PHENIX, using only the UMAP space over-smooths the data (S4 Section), this demonstrates the necessity of PCA as initialization for diffusion on UMAP space. Only UMAP does not capture well-high dimensionality of the data. Many studies have shown that PCA helps UMAP to obtain a more detailed local and global structure [5,6].

In general, MAGIC needs to be used with low *knn* and *t* values, and less principal component dimensions for a less over-smoothed imputation. Contrastingly, sc-PHENIX is more robust to not induce over-smoothing by increasing PCA dimensions, *knn* and *t*. Thus, sc-PHENIX using PCA-UMAP space captures more of the variability and portrays a more accurate graph, without distorting the recovered expression driven by a diffusion on PCA space.

As already mentioned in the previous section of this work, the MAGIC (diffusion on PCA space) will over-smoothing scRNA-seq data because of the artifact that renders a connection of densities from distinct cell phenotypes. This makes distinct cell phenotypes similar and imputation recovers similar expressions among these distinct cell phenotypes. We only show in this section a small number of cell phenotypes. However, with MAGIC the over-smoothing will increase as more distinct cell phenotypes are in an experiment due to a more chances of connections among distinct cell phenotypes that are near to each other in the high dimensional PCA space. However, sc-PHENIX mitigates this artifact effect.

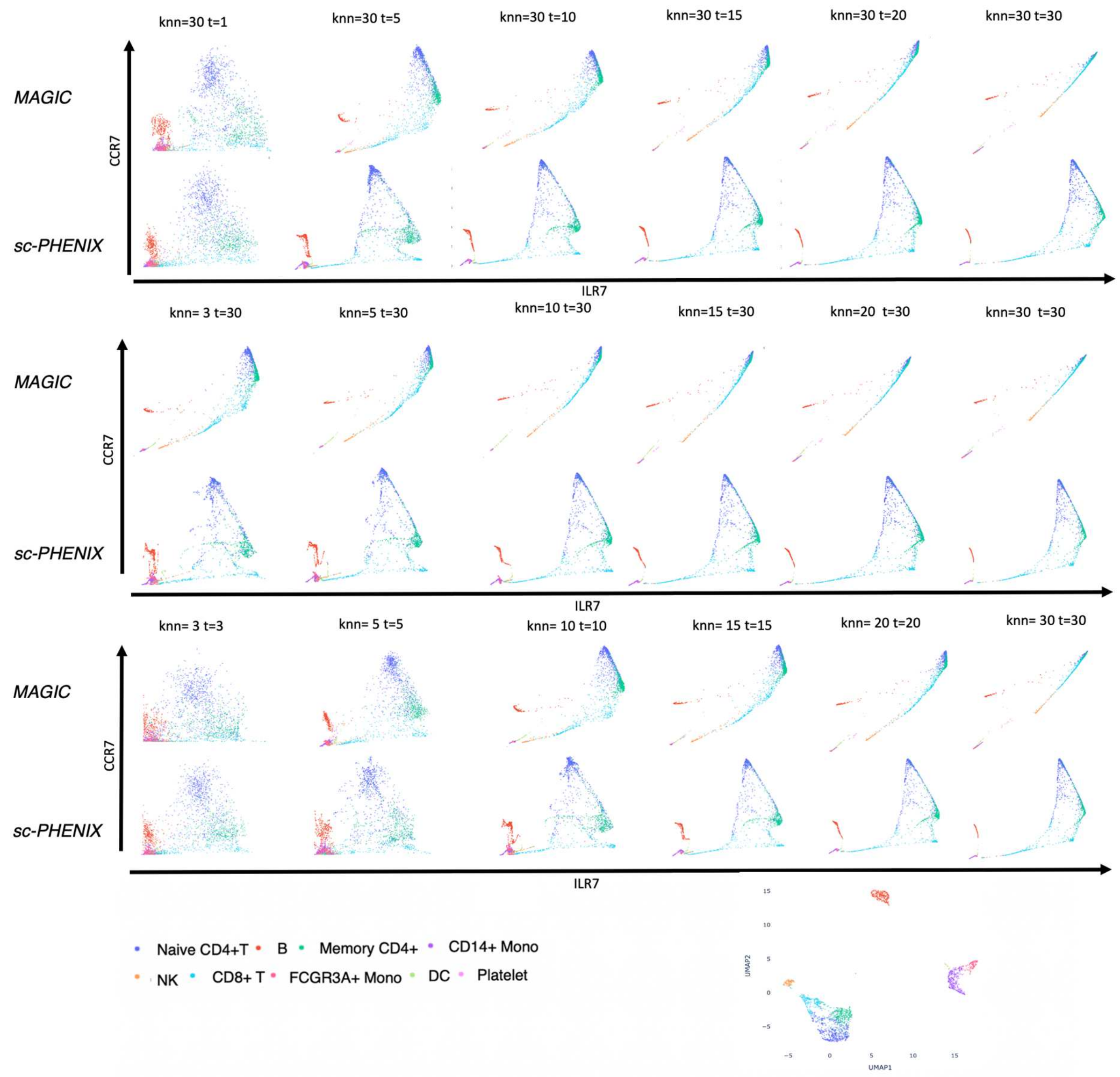

Fig II Effect on the gene-gene interactions using different values of the parameter *knn* and *t* used on MAGIC and sc-PHENIX

Each dot represents a cell plotted based on their imputed gene expression of IL7R and CCR7 by MAGIC and sc-PHENIX, As *knn* and *t* increases the MAGIC's recovered IL7R-CCR7 interaction, it starts to recover IL7R and CCR7 values to unrelated cell phenotypes. Also the continuum structure is lost by distorting the dynamic transition of naive to memory CD4+T cells. With sc-PHENIX, the IL7R-CCR7 shape is robust along all combinations of diffusion parameters and continuum structure is captured well by matening the dynamic transition of naive to memory CD4+T cells. In below: UMAP plot of PBMC cell phenotypes.

1. Lee MS, Hanspers K, Barker CS, Korn AP, McCune JM. Gene expression profiles during human CD4+ T cell differentiation. International Immunology. 2004 Jun 21;16(8):1109–24.

2. van Dijk D, Sharma R, Nainys J, Yim K, Kathail P, Carr AJ, et al. Recovering Gene Interactions from Single-Cell Data Using Data Diffusion. Cell. 2018 Jul;174(3):716-729.e27.

3. Tian, Babor, Lane, Schulten, Patil, Seumois, et al. Unique phenotypes and clonal expansions of human CD4 effector memory T cells re-expressing CD45RA. Nature Communications. 2017 Nov 13;8(1):1–13.

4. Sheikh A, Abraham N. Interleukin-7 Receptor Alpha in Innate Lymphoid Cells: More Than a Marker. Frontiers in Immunology. 2019 Jan 1;0.

5. Kobak D, Linderman GC. Initialization is critical for preserving global data structure in both t-SNE and UMAP. Nature Biotechnology. 2021 Feb;39(2):156–7.

6. Sakaue S, Hirata J, Kanai M, Suzuki K, Akiyama M, Lai Too C, et al. Dimensionality reduction reveals fine-scale structure in the Japanese population with consequences for polygenic risk prediction. Nature Communications. 2020 Mar 26;11(1).
