# Supplementary material for: Diffusion on PCA-UMAP Manifold: The Impact of Data Structure Preservation to Denoise High-Dimensional Single-Cell RNA Sequencing Data
Source: Biology (Basel). 2024 Jul 9;13(7):512. doi: 10.3390/biology13070512 (PMC11274112; doi:10.3390/biology13070512)
Supplement: Supplementary file 1 [file biology-13-00512-s001.zip › SM/Supple_ Sections/Section S6 Effect of sparcity in embeddings.pdf]

Section S6. Corrupted image digits data visualized using PCA, UMAP (with PCA initialization) and PHATE through different rates of induced dropout-events.

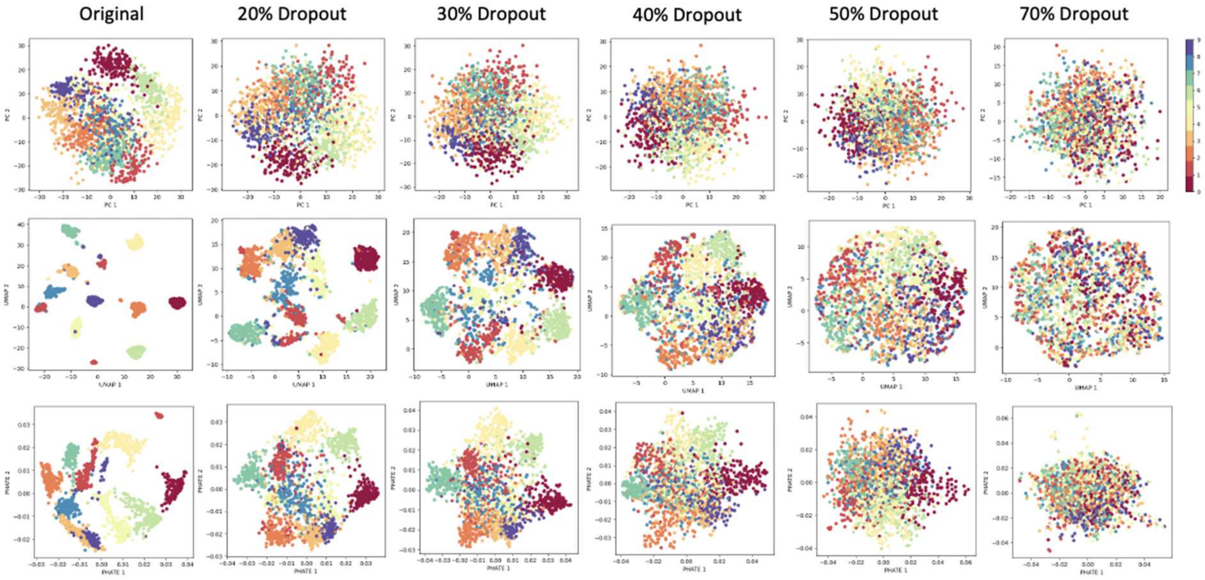

Fig A. Corrupted image digits data visualized using PCA (First row), UMAP (second row) and PHATE (third row) through different rates of induced dropout-events. The UMAP and PHATE had an initialization of 50 PCs.

The data set that we used is the image digit data set [1]. The intention of this section is to depict how different rates of induced zeros values can affect the nearness of distinct clusters and the structure of the data. We observed that loss of the local structure as the rate of induced zeros increases. Therefore, distinct digit clusters are getting closer, losing the information about the heterogeneity in an unsupervised visualization. It seems that PHATE that uses a diffusion approach seems to be more affected to the rate of induced zeros values rather than UMAP. Probability, this indicates that UMAP is more adequate for spare data.

1.Dua, D. and Graff, C. (2019). UCI Machine Learning Repository [http://archive.ics.uci.edu/ml]. Irvine, CA: University of California, School of Information and Computer Science.
