# Supplementary material for: Diffusion on PCA-UMAP Manifold: The Impact of Data Structure Preservation to Denoise High-Dimensional Single-Cell RNA Sequencing Data
Source: Biology (Basel). 2024 Jul 9;13(7):512. doi: 10.3390/biology13070512 (PMC11274112; doi:10.3390/biology13070512)
Supplement: Supplementary file 1 [file biology-13-00512-s001.zip › SM/Supple_ Sections/Section S7 Validating Over-Imputation in MAGIC Using k-Means_ Addressing Potential Bias in Differential Expression Analysis with EMD.pdf]

The importance of this section is to corroborate whether the over-imputation of MAGIC is a side effect due to a biased differential expression cluster analysis using EMD (Earth mover's distance). This analysis may be biased because the clusters obtained from the 3D-PCA plot of the MAGIC-imputed data have more samples and are closer to each other. Having more samples might mask the heterogeneity of the data and not be because of over-smoothing of MAGIC. To corroborate the previous, we applied k-means clustering to the 3D-PCA space from the imputed data to force to detect 11 clusters see Fig A and S12 Fig (interactive plot). Then we applied differential expression cluster analysis using EMD (Fig B). As we can observe, it is that based on the dendrogram data shows that there are 2 main clusters

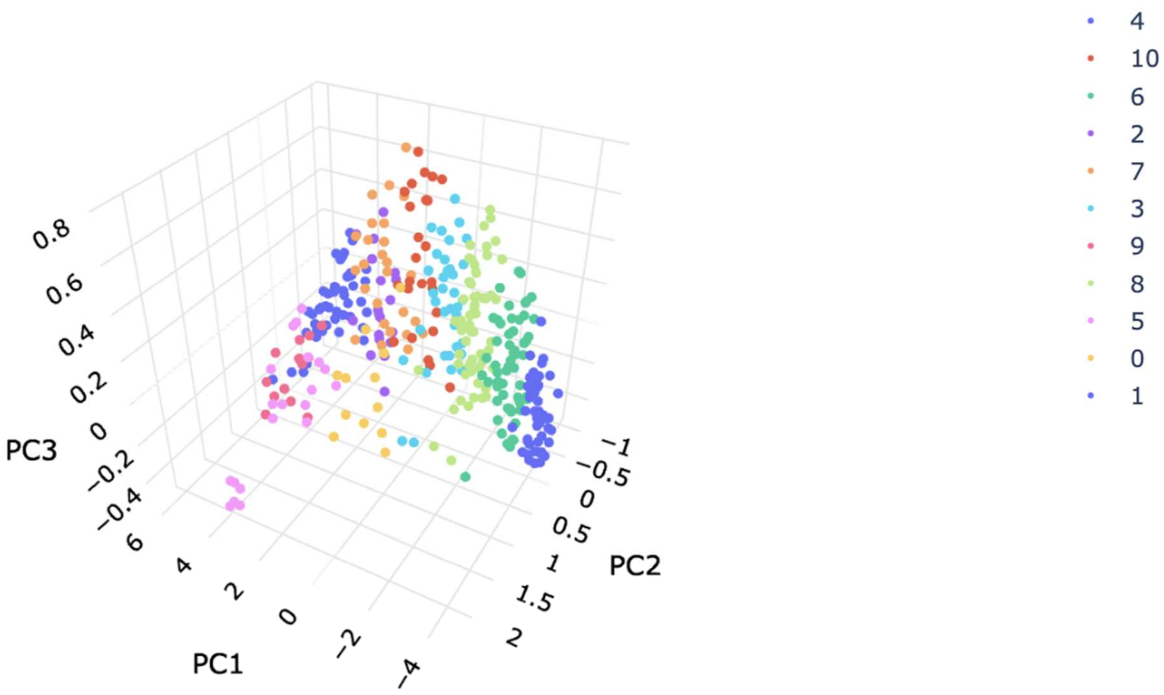

Fig A 3D-PCA plot of the imputed data by MAGIC

The labes are the eleven clusters detected by k means algorithm

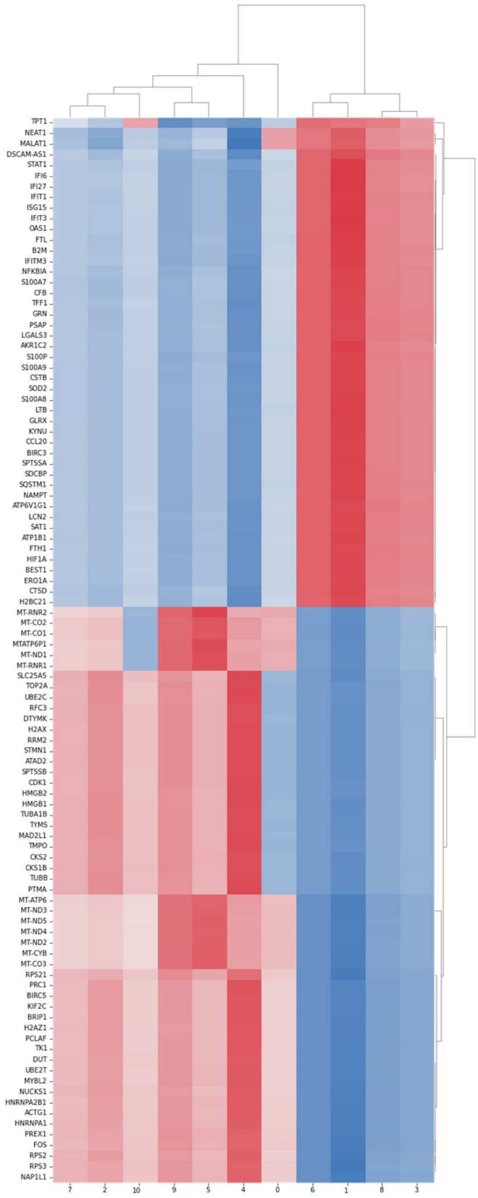

Fig B Differential expression by 11 k means clusters

Heatmap of DEG (Differential expressed genes) using EMD score (red and blue-ish colored), DEG (rows) for each HDBSCAN clusters (columns in numbers)
