# Supplementary material for: Diffusion on PCA-UMAP Manifold: The Impact of Data Structure Preservation to Denoise High-Dimensional Single-Cell RNA Sequencing Data
Source: Biology (Basel). 2024 Jul 9;13(7):512. doi: 10.3390/biology13070512 (PMC11274112; doi:10.3390/biology13070512)
Supplement: Supplementary file 1 [file biology-13-00512-s001.zip › SM/Supple_ Sections/Section S8 Comparative Analysis of Gene-Gene Interactions and 3D PCA Plots from sc-PHENIX and MAGIC Imputations on MCF7 MCTS Dataset.pdf]

Gene-gene interaction and 3D PCA plots of the sc-PHENIX and MAGIC imputation with MCF7 MCTS dataset.

Here, we show 3D-PCA and the FN1-CDH1-VIM interaction plots, of imputed the Multicellular Cancer Tumor Spheroids of breast cancer cell line MCF7 dataset (MCF7 MCTS) [1] WITH sc-PHENIX and MAGIC. This in order to see the differences of imputation via diffusion on PCA-UMAP space (sc-PHENIX) and PCA (MAGIC) space. On these plots (Fig A, B and C), recovered gene expression is visualized. In Fig A, we observe with sc-PHENIX the cluster “2” presents an over representation of VEFGA gene expression, indicating that this cell subpopulation could be a necrotic state. Also, in Fig D we observed that the expression of the MT-ND1 gene is in the same population. Furthermore in the main text, in Fig 10A (heatmap of highlighted genes with sc-PHENIX), the MT-genes are over-represented on the cluster “2”. Here, observing the consistency of a necrotic state. However, with MAGIC the over representation of VEFGA.

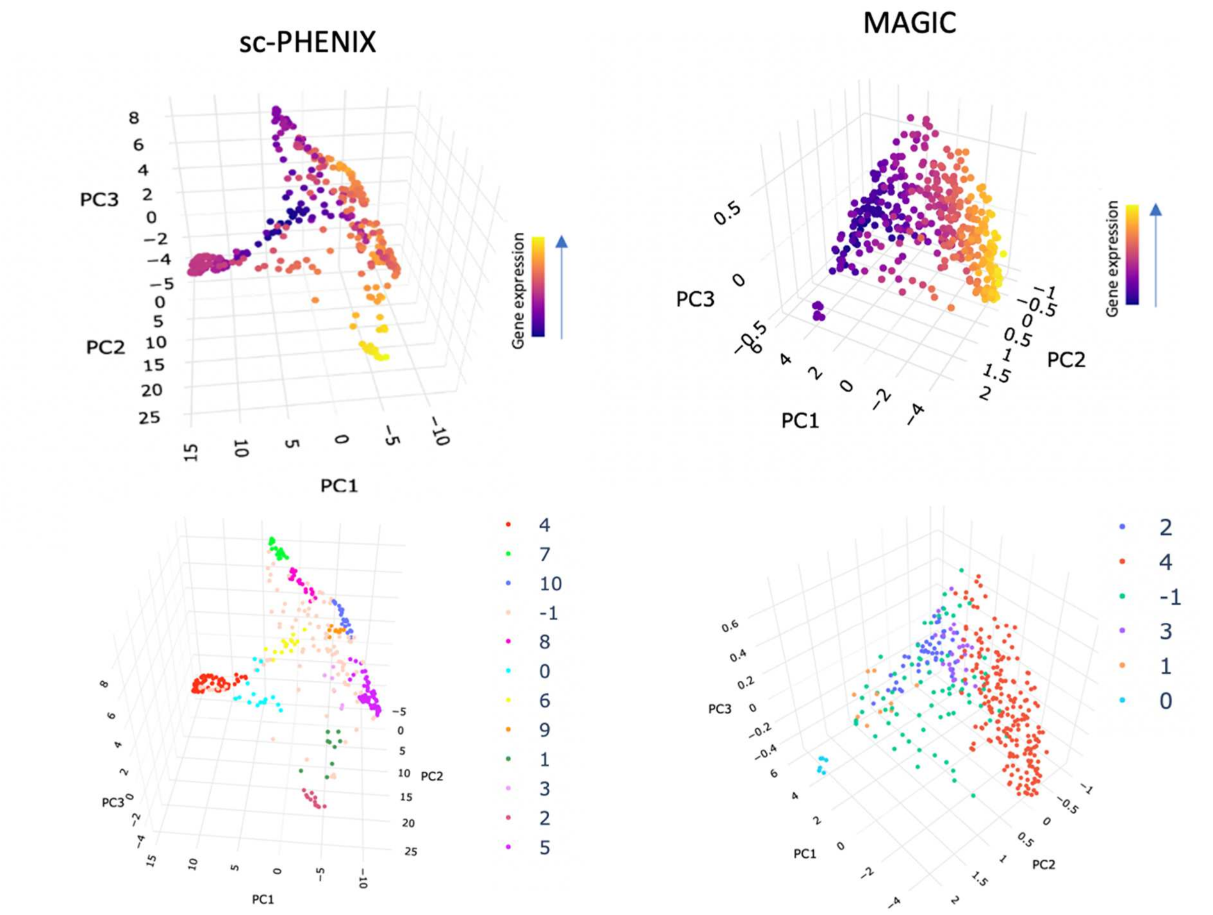

Fig A Expression of the recovered VEFGA gene by MAGIC and sc-PHENIX
Up: VEFGA expression on the 3D PCA plot of the imputed data. Down: Dense clusters(detected by HDBSCAN) on the 3D PCA plot of the imputed data.

In Fig B, we can visualize that expression of the MKI79 in the VIM-CDH1 interaction. With MAGIC we observed a distortion of the continuum structure reflected on the magnitude of gene expression of this interaction. VIM and CDH1 are involved in the canonical feature in EMT (epithelial

mesenchymal transition), VIM is downregulated when CDH1 is upregulated. VIM is a mesenchymal marker, CDH1 is an epithelial marker, and MLI79 is a proliferation marker. We observe that in Fig B with MAGIC, at high levels of CDH1 there is https://www.pnas.org/doi/10.1073/pnas.1519197113. With sc-PHENIX, we observe that there is a mesenchymal cell type that has high VIM expression and low CDH1 expression, where has strong over-representation of MKI79, this Mesenchymal stem cells derived from breast cancer tissue promote the proliferation for this specific MCF7 cell line (https://pubmed.ncbi.nlm.nih.gov/24260049/).

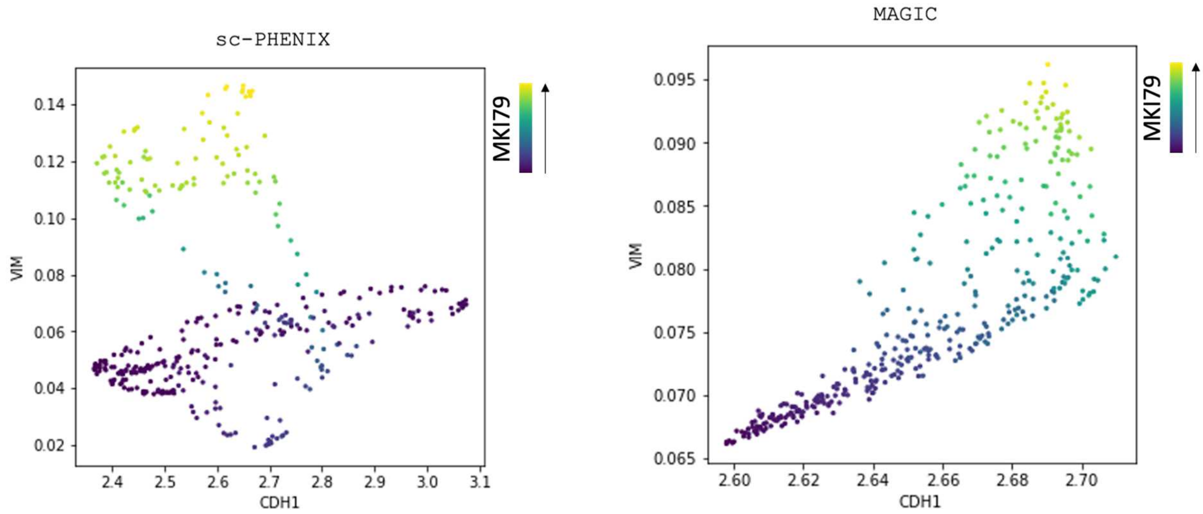

Fig B VIM-CDH1 interaction with sc-PHENIX and MAGIC. MKI67

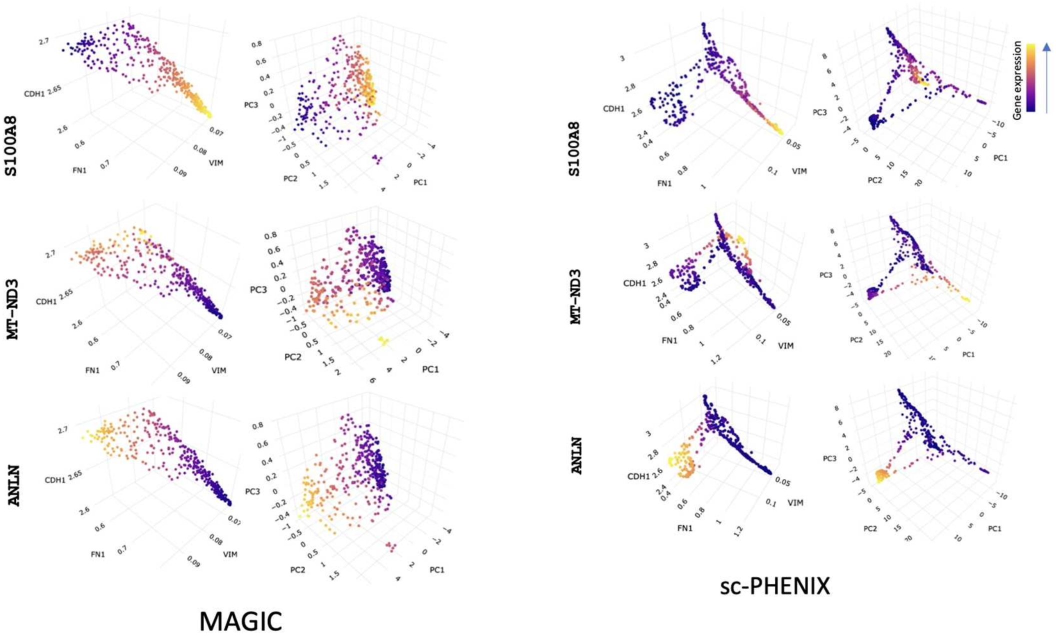

Fig C Gene-gene interaction with sc-PHENIX and MAGIC. FN1- CDH1-VIM

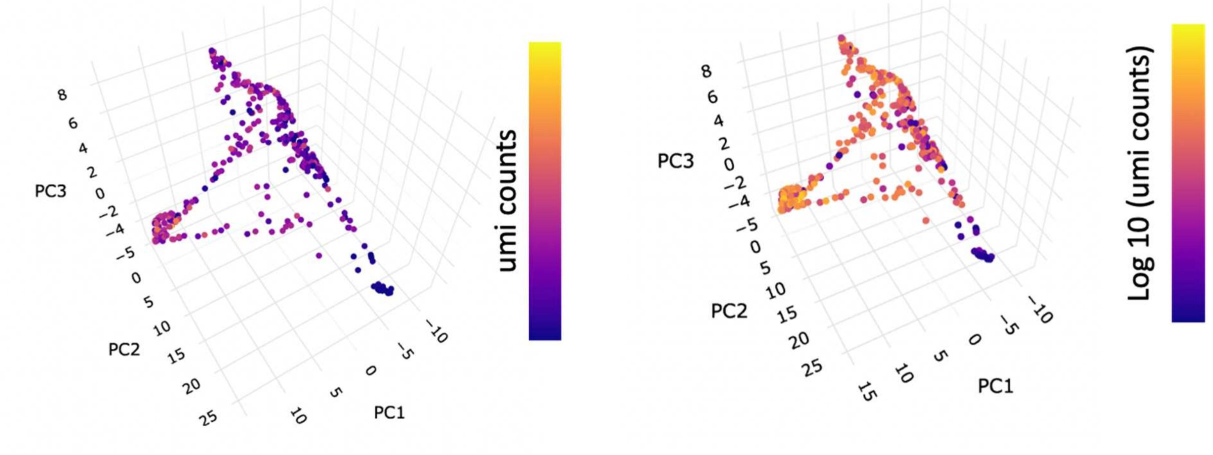

Fig D library size embedded in the 3D PCA projection of the imputed data with sc-PHENIX

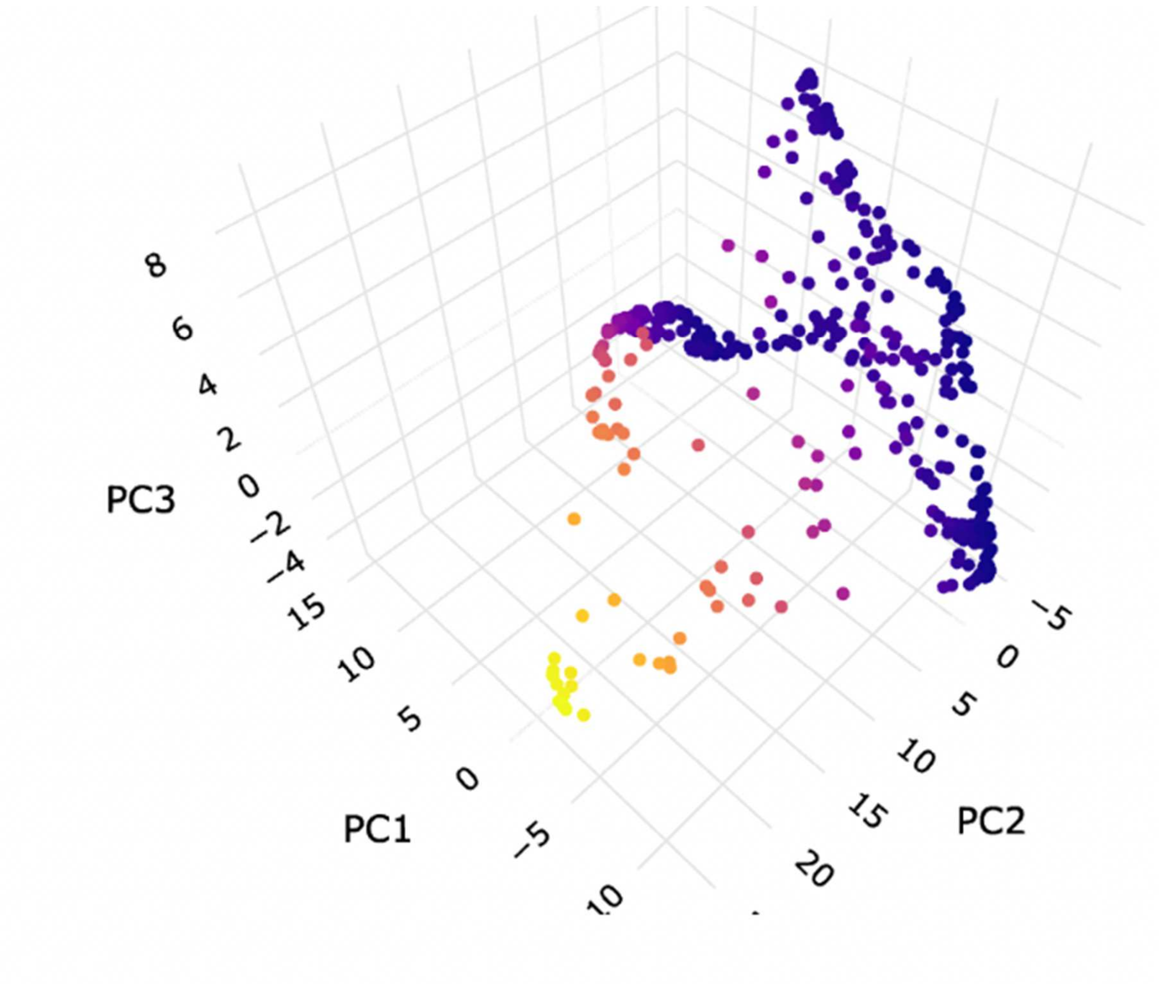

Fig D MT-ND1 recover expression with sc-PHENIX.

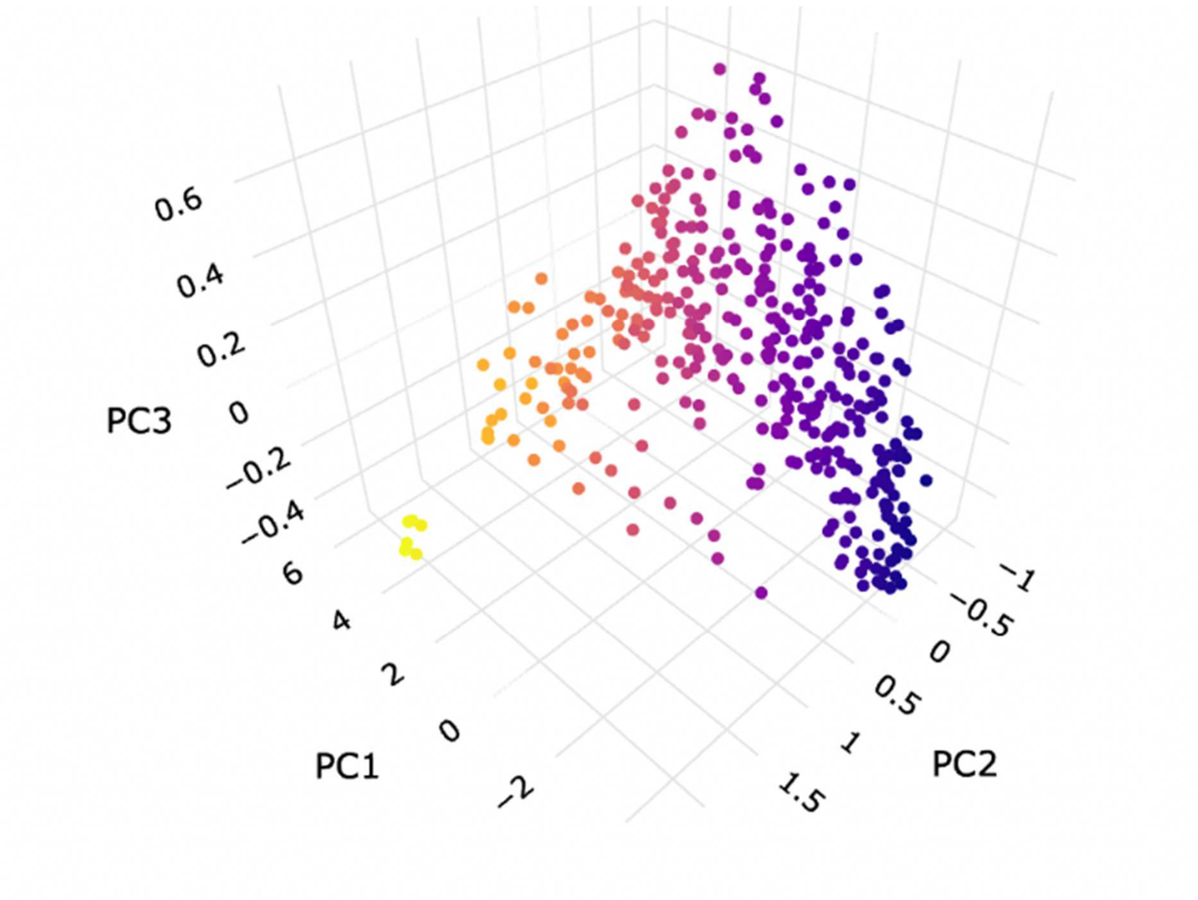

Fig E MT-ND1 recover expression with MAGIC
