# Supplementary material for: Diffusion on PCA-UMAP Manifold: The Impact of Data Structure Preservation to Denoise High-Dimensional Single-Cell RNA Sequencing Data
Source: Biology (Basel). 2024 Jul 9;13(7):512. doi: 10.3390/biology13070512 (PMC11274112; doi:10.3390/biology13070512)
Supplement: Supplementary file 1 [file biology-13-00512-s001.zip › SM/Supple_ Sections/Section S9 knn smoothing in PBMC and neuronal datasets_.pdf]

In this section in particular, we discussed the results of the knn-smoothing [1] applied on the PBMC and neuronal datasets. knn-smoothing is one of the methods (among MAGIC and SAVER) that outperforms in the analysis post imputation in benchmarkings [2,3]. The knn-smoothing algorithm is designed to reduce noise by aggregating information from similar cells (neighbors) in a computationally efficient and statistically tractable manner. This algorithm is based on the observation that across protocols, the technical noise exhibited by UMI-filtered scRNA-Seq data closely follows Poisson statistics [1].

Before discussing the results about knn-smoothing, the biological implication of the gene-gene interactions in PBMC data mentioned are showed in S3 Section. In S3 Section, we discuss and show the results of the imputed PBMC data with MAGIC, SAVER and sc-PHENIX. In S3 Section with sc-PHENIX revels information about transition of naive to a memory T+ cell state differentiation (this transition involves a CCR7 downregulation), and the detection of a small CD45RA+CCR7-effector memory T cells that re-expresses CD45RA+ population with sc-PHENIX. Also, sc-PHENIX captures the continuum structure better and does not compromise this continuum by local data structure loss (S1 Section).

In contrast, with knn-smoothing, we observe that the CCR7-IL7R and IL7R-PTPRC interactions imputed with knn-smoothing present distortion (over-smoothing) in Fig A and B, we can observe that local structure is not preserve in Fig B. If we compared the imputed gene-gene interactions obtained from knn-smoothing (Fig B) and PHENIX, PHENIX does a better job regarding preserving data sturcture (see, CCR7-IL7R and IL7R-PTPRC recovered by sc-PHENIX in S1 Section).

In Fig A, there is down regulation of CCR7 involved in the transition of a naive to a memory CD4+T cell phenotype, capturing the continuum. However, at first glance in Fig A, it seems to be one single and not-well-structured ramification from the naive to memory CD4+T cells. With knn-smoothing, granular details of other subpopulations can not be visualized compared to sc-PHENIX (Fig IC in S1 Section). As a consequence, the small CD45RA+CCR7-effector memory T cells that re-expresses CD45RA+ population (that expresses more FOXP3) is not present (Fig B) but is present with sc-PHENIX (S1 Section).

Also, knn-smoothing results compared to MAGIC or sc-PHENIX ones, there is not a defined continuum structure it seems to be shattered. Probably this due to its aggregation of information methodology based on a PCA space. It is known that local structure can not be captured well with PCA in sc-RNA-seq data (see, introduction).

In Fig C for the neuronal dataset, we can observe that knn-smoothing imputation does a great job in keeping gene markers within their respective cell phenotype. However in a dataset such as PBMC the study of cell transition phenotypes could lead to wrong results. The method sc-PHENIX does

contemplate avoiding over-smoothing and capture a well representation of continuum structure. This in order to find rare phenotypes based on gene-gene interactions.

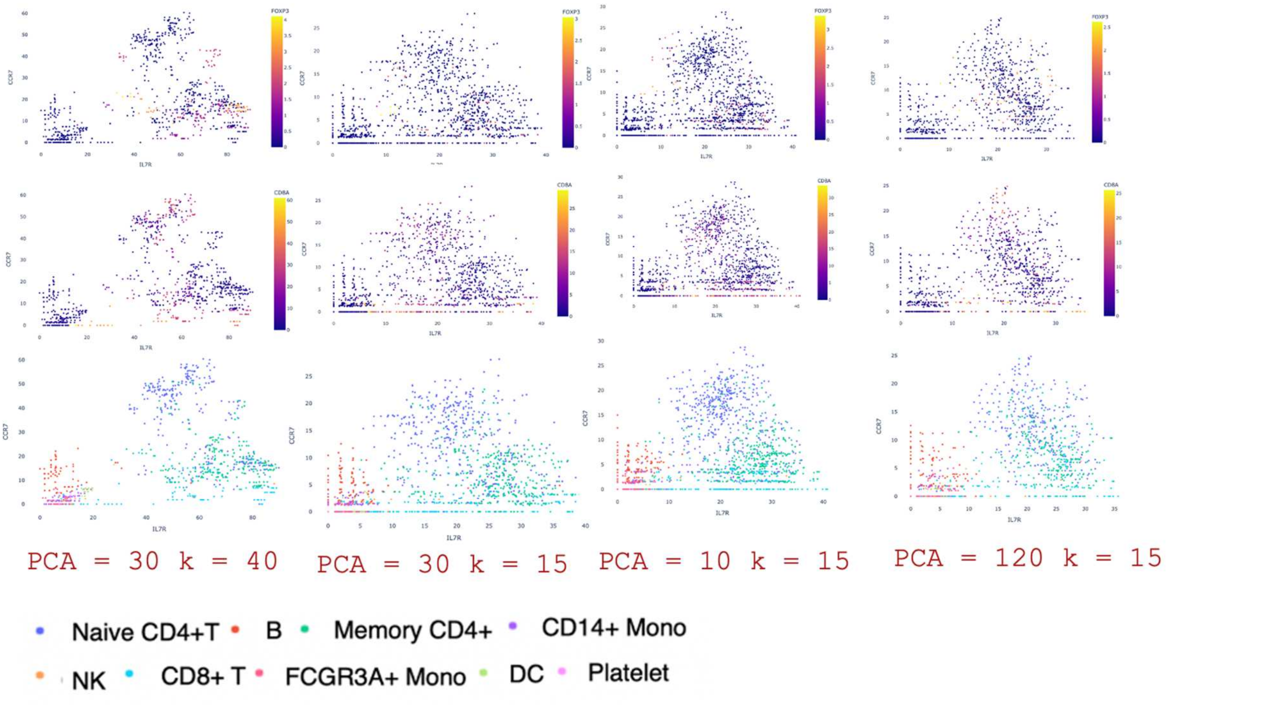

Fig A IL7R-CCR7 interaction of the non-imputed 3k PBMC dataset and the imputed by knn-smoothing, distinct PBMC cell phenotypes are shown below.

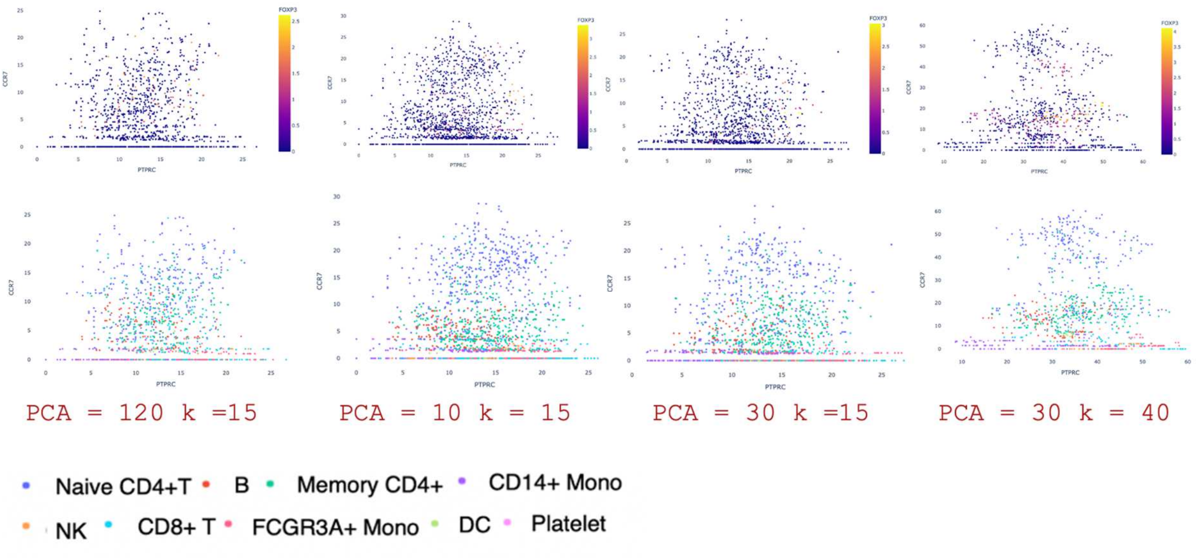

Fig B IL7R-PTPRC interaction of the non-imputed 3k PBMC dataset and the imputed by knn-smoothing, distinct PBMC cell phenotypes are shown below. CD45RA is also known as PTPRC)

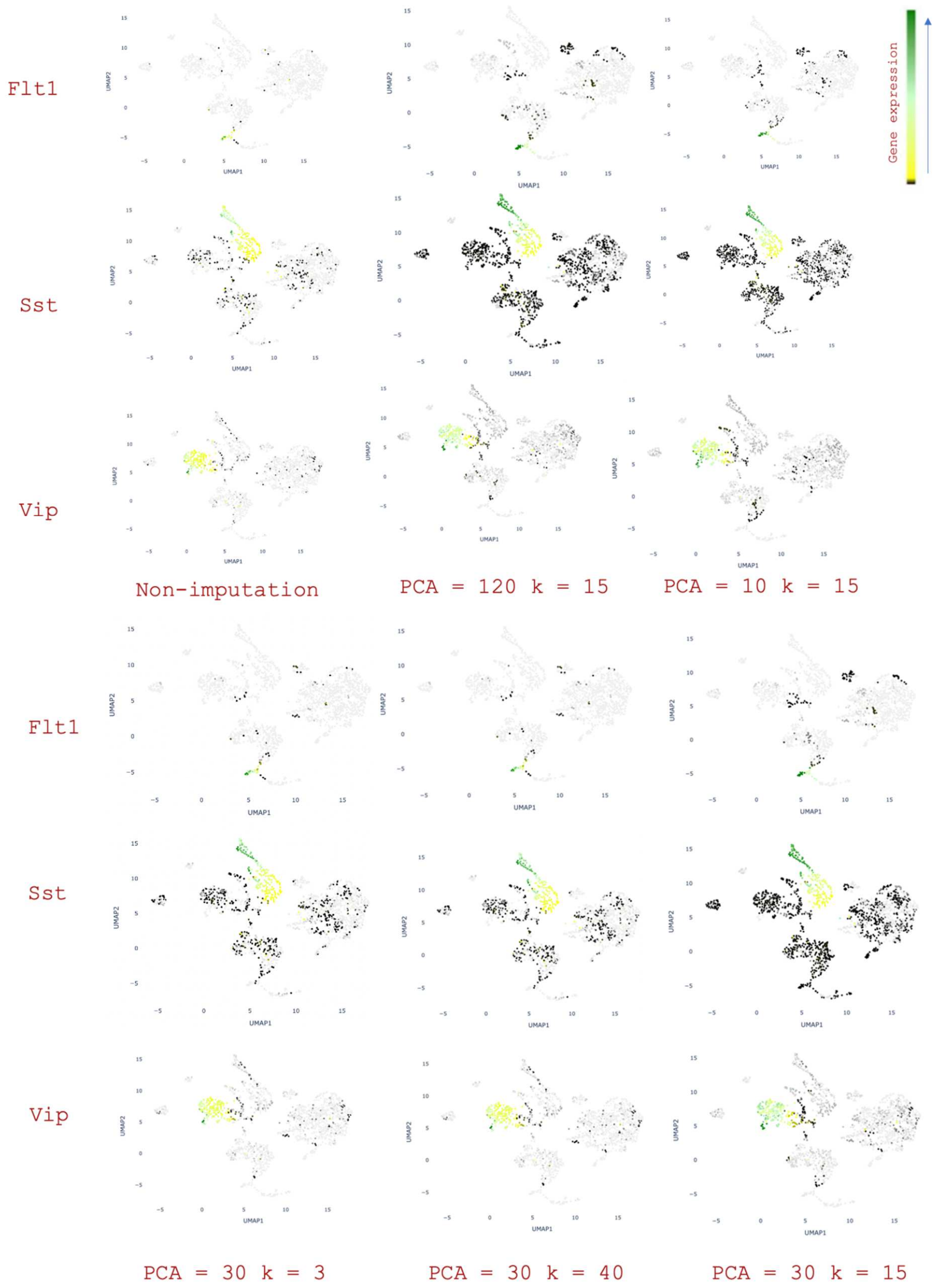

Fig C Imputation of the adult mouse visual cortex using knn-smoothing
Recovered Flt1, Sst and Vip expressions are visualized on UMAP projection of the adult mouse visual cortex cells dataset. The UMAP projection is based on Fig 6.

References

1. Wagner F, Yan Y, Yanai I. K-nearest neighbor smoothing for high-throughput single-cell RNA-Seq data. bioRxiv. 2018. p. 217737. doi:10.1101/217737

2. Hou W, Ji Z, Ji H, Hicks SC. A systematic evaluation of single-cell RNA-sequencing imputation methods. Genome Biol. 2020;21: 218.

3. Patruno L, Maspero D, Craighero F, Angaroni F, Antoniotti M, Graudenzi A. A review of computational strategies for denoising and imputation of single-cell transcriptomic data. Brief Bioinform. 2021;22. doi:10.1093/bib/bbaa222

Commands used for kNN-smoothing

```
python knn-smoothing-master/knn_smooth.py -f expression_matrix.txt -o expression_matrix_PCA10_k15.cvs -d 10 -k 15
 python knn-smoothing-master/knn_smooth.py -f expression_matrix.txt -o expression_matrix_PCA30_k15.cvs -d 30 -k 15
 python knn-smoothing-master/knn_smooth.py -f expression_matrix.txt -o expression_matrix_PCA120_k15.cvs -d 120 -k 15
 python knn-smoothing-master/knn_smooth.py -f expression_matrix.txt -o expression_matrix_PCA30_k3.cvs -d 30 -k 3
 python knn-smoothing-master/knn_smooth.py -f expression_matrix.txt -o expression_matrix_PCA30_k40.cvs -d 30 -k 40
```
