# Supplementary figures and images for: Diffusion on PCA-UMAP Manifold: The Impact of Data Structure Preservation to Denoise High-Dimensional Single-Cell RNA Sequencing Data
Source: Biology (Basel). 2024 Jul 9;13(7):512. doi: 10.3390/biology13070512 (PMC11274112; doi:10.3390/biology13070512)

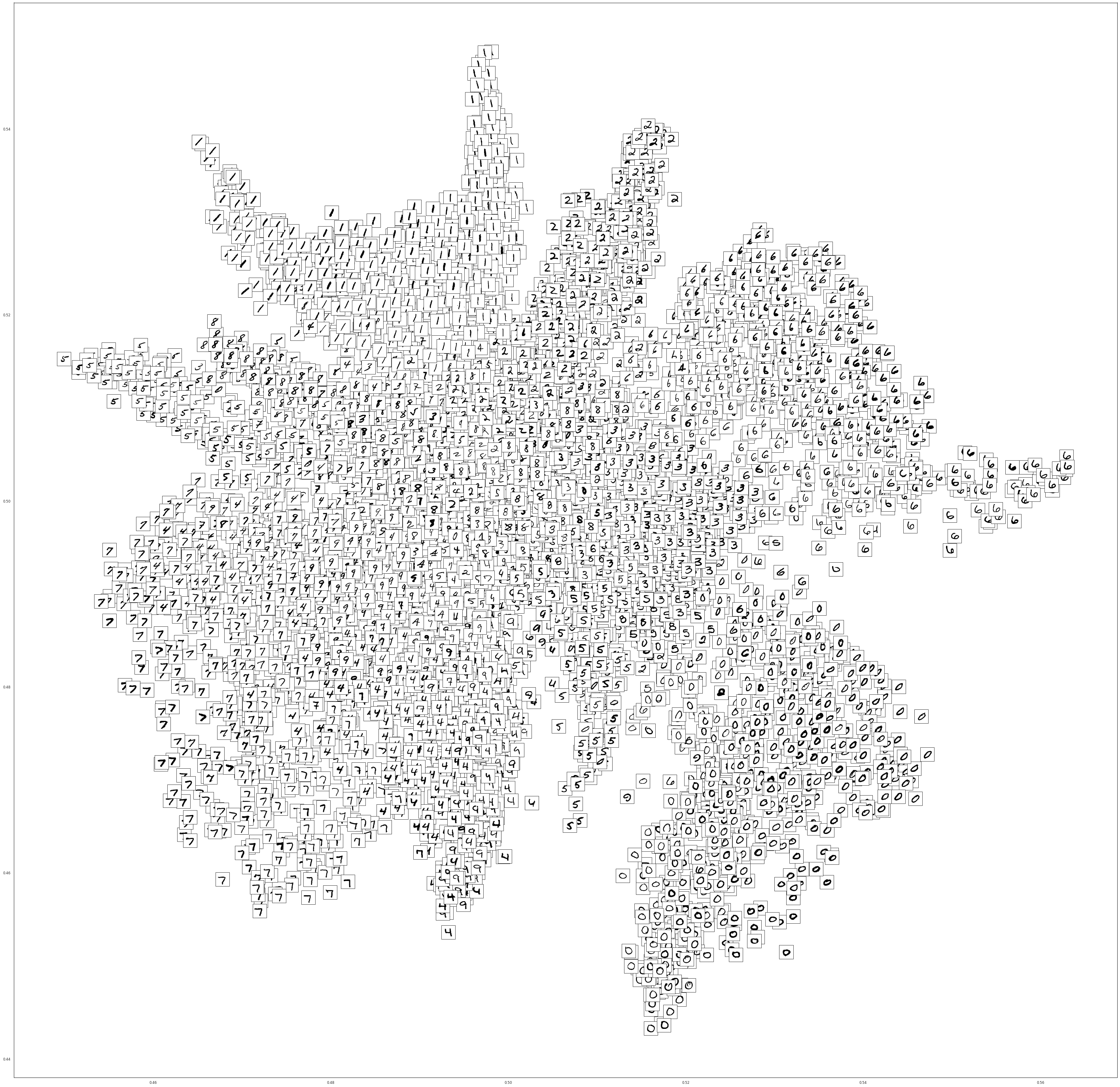

Supplement: Supplementary file 1 [file biology-13-00512-s001.zip › SM/Supple_ Figures/FIG S3 images in manifols mt pca init.png]

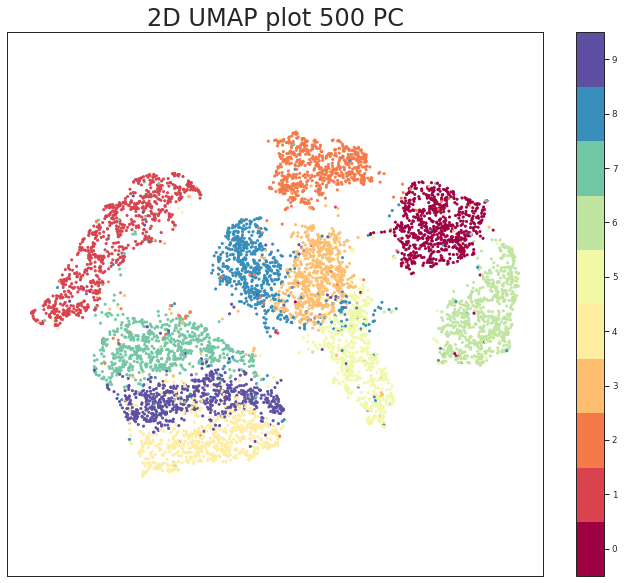

Supplement: Supplementary file 1 [file biology-13-00512-s001.zip › SM/Supple_ Figures/FIG S9 UMAP MNIST POINTS.png]

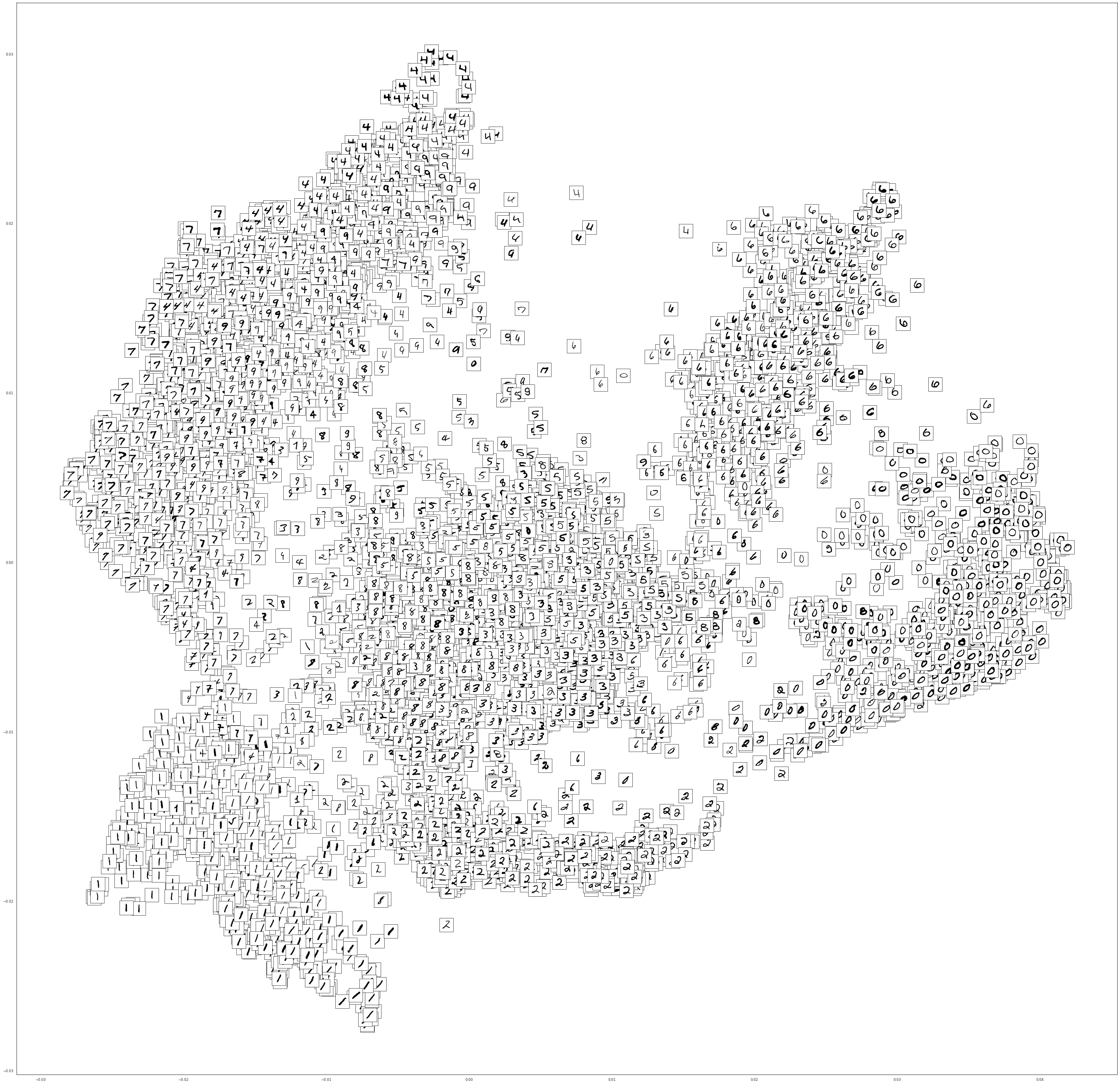

Supplement: Supplementary file 1 [file biology-13-00512-s001.zip › SM/Supple_ Figures/FIG S8 2D PHATE PLOT MNIST images on embedding.png]

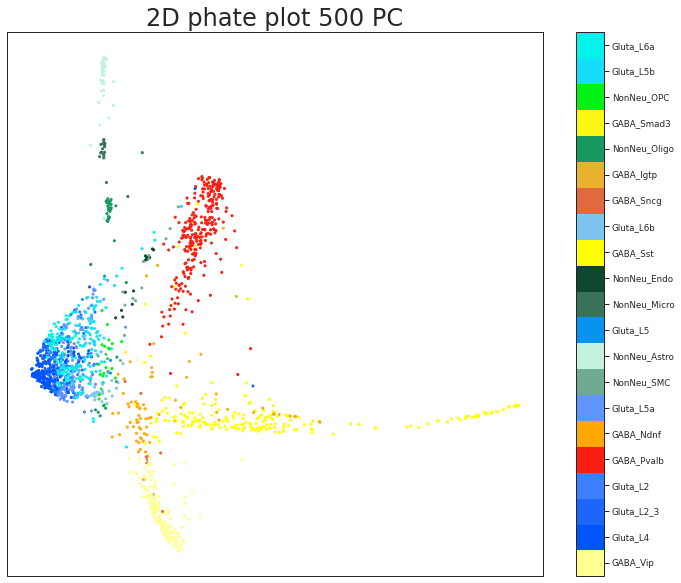

Supplement: Supplementary file 1 [file biology-13-00512-s001.zip › SM/Supple_ Figures/FIG S6 PHATE plot neuronal.png]

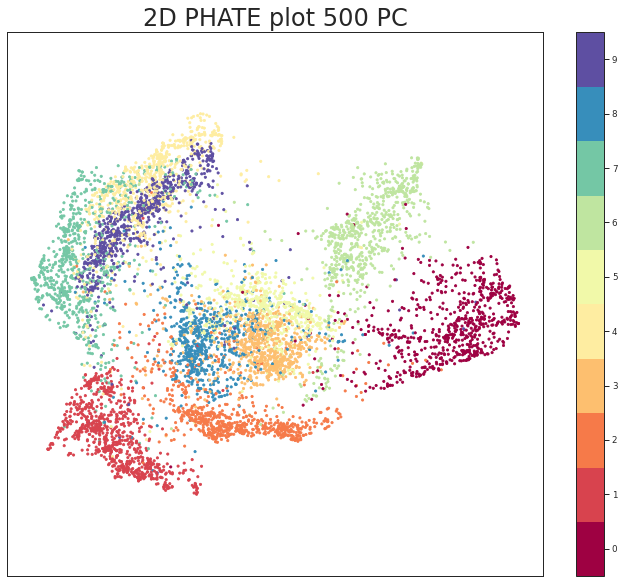

Supplement: Supplementary file 1 [file biology-13-00512-s001.zip › SM/Supple_ Figures/FIG S7 2D PHATE PLOT MNIST POINTS.png]

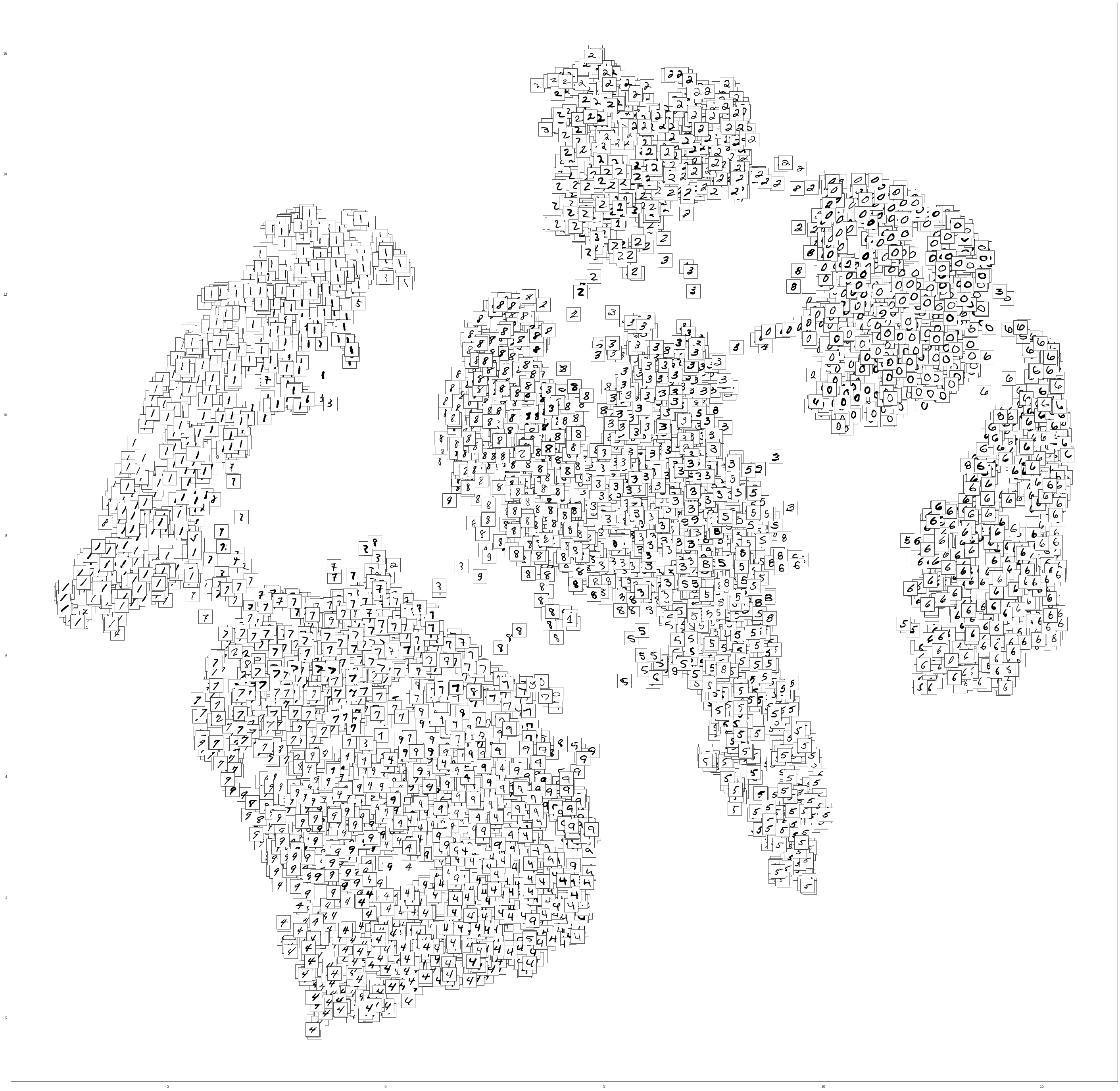

Supplement: Supplementary file 1 [file biology-13-00512-s001.zip › SM/Supple_ Figures/FIG S10 UMAP MNIST imagen on embedding.png]

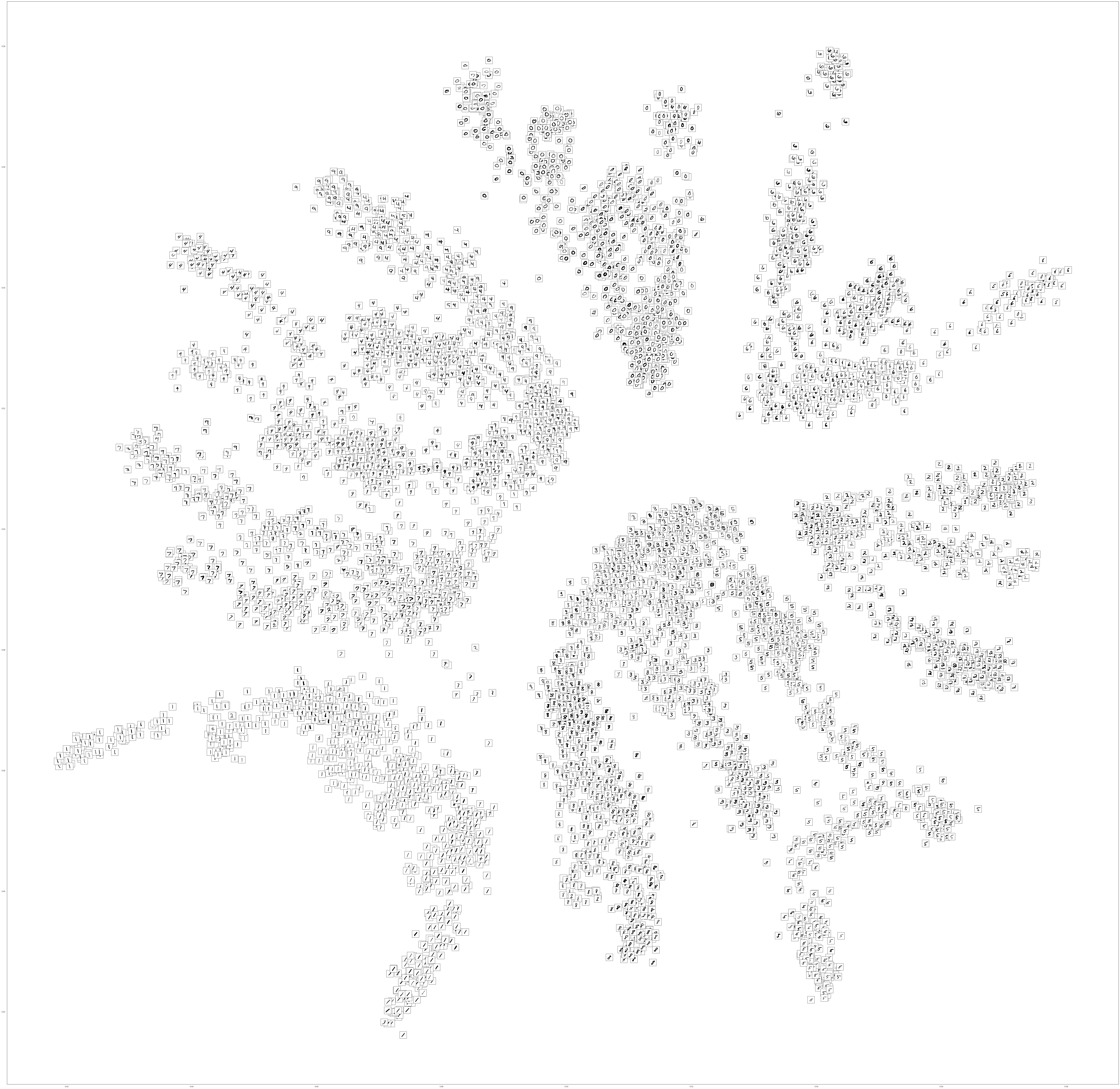

Supplement: Supplementary file 1 [file biology-13-00512-s001.zip › SM/Supple_ Figures/FIG S4 images in manifold pca-umap mt.png]
