# Supplementary material for: Diffusion on PCA-UMAP Manifold: The Impact of Data Structure Preservation to Denoise High-Dimensional Single-Cell RNA Sequencing Data
Source: Biology (Basel). 2024 Jul 9;13(7):512. doi: 10.3390/biology13070512 (PMC11274112; doi:10.3390/biology13070512)
Supplement: Supplementary file 1 [file FIG S11 More umap plots of 2d umap and markers.pdf › SM/Supple_ Figures/FIG S11 More umap plots of 2d umap and markers.pdf]

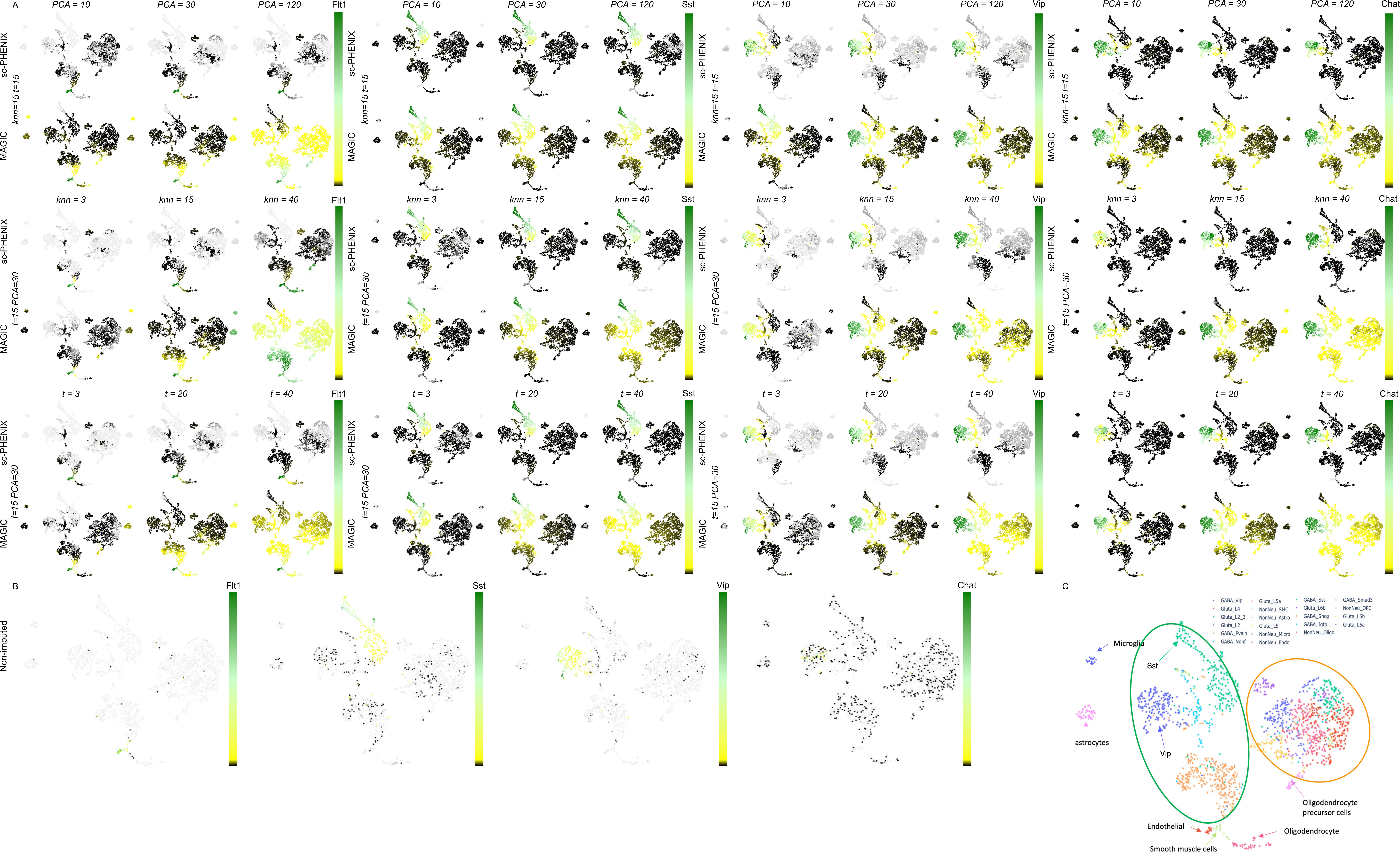
A
PCA = 10
PCA = 30
PCA = 120
Flt1
Sst
Vip
Chat
sc-PHENIX
MAGIC
knn=15 t=15
knn = 3
knn = 15
knn = 40
t=15 PCA=30
t = 3
t = 20
t = 40
B
Non-imputed
C
GABA_Vip
Gluta_L4
Gluta_L2_3
Gluta_L2
GABA_Pvalb
GABA_Ndnf
Gluta_L5a
NonNeu_SMC
NonNeu_Astro
Gluta_L5
NonNeu_Micro
NonNeu_Endo
GABA_Sst
Gluta_L6b
GABA_Sncg
GABA_Igtp
NonNeu_Oligo
GABA_Smad3
NonNeu_OPC
Gluta_L5b
Gluta_L6a
Microglia
Sst
astrocytes
Vip
Oligodendrocyte precursor cells
Endothelial
Oligodendrocyte
Smooth muscle cells
